# Cholic and deoxycholic acids induce mitochondrial dysfunction, impaired biogenesis and autophagic flux in skeletal muscle cells

**DOI:** 10.1186/s40659-023-00436-3

**Published:** 2023-06-08

**Authors:** Johanna Abrigo, Hugo Olguín, Franco Tacchi, Josué Orozco-Aguilar, Mayalen Valero-Breton, Jorge Soto, Mauricio Castro-Sepúlveda, Alvaro A. Elorza, Felipe Simon, Claudio Cabello-Verrugio

**Affiliations:** 1 Laboratory of Muscle Pathology, Fragility and Aging, Department of Biological Sciences, Faculty of Life Sciences, Universidad Andres Bello, Santiago, Chile; 2 Millennium Institute on Immunology and Immunotherapy, Faculty of Life Sciences, Universidad Andres Bello, Santiago, Chile; 3 Center for the Development of Nanoscience and Nanotechnology (CEDENNA), Universidad de Santiago de Chile, Santiago, Chile; 4 Laboratory of Tissue Repair and Adult Stem Cells, Department of Cellular and Molecular Biology, Faculty of Biological Sciences, Pontificia Universidad Católica de Chile, Santiago, Chile; 5 Laboratorio de Ensayos Biológicos (LEBi), Universidad de Costa Rica, San José, Costa Rica; 6 Facultad de Farmacia, Universidad de Costa Rica, San José, Costa Rica; 7 Millennium Institute on Immunology and Immunotherapy, Departamento de Genética Molecular y Microbiología, Facultad de Ciencias Biológicas, Pontificia Universidad Católica de Chile, Santiago, Chile; 8 Exercise Physiology and Metabolism Laboratory, School of Kinesiology, Faculty of Medicine, Finis Terrae University, Santiago, Chile; 9 Institute of Biomedical Sciences, Faculty of Medicine, and Faculty of Life Sciences, Universidad Andres Bello, Santiago, Chile; 10 Millennium Nucleus of Ion Channel-Associated Diseases (MiNICAD), Universidad de Chile, Santiago, Chile; 11 Laboratory of Integrative Physiopathology, Department of Biological Sciences, Faculty of Life Sciences, Universidad Andres Bello, Santiago, Chile

**Keywords:** Mitochondrial dysfunction, Biogenesis, Mitophagy, Skeletal muscle, Oxygen consumption, Bile acids

## Abstract

**Background:**

Skeletal muscle is sensitive to bile acids (BA) because it expresses the TGR5 receptor for BA. Cholic (CA) and deoxycholic (DCA) acids induce a sarcopenia-like phenotype through TGR5-dependent mechanisms. Besides, a mouse model of cholestasis-induced sarcopenia was characterised by increased levels of serum BA and muscle weakness, alterations that are dependent on TGR5 expression. Mitochondrial alterations, such as decreased mitochondrial potential and oxygen consumption rate (OCR), increased mitochondrial reactive oxygen species (mtROS) and unbalanced biogenesis and mitophagy, have not been studied in BA-induced sarcopenia.

**Methods:**

We evaluated the effects of DCA and CA on mitochondrial alterations in C_2_C_12_ myotubes and a mouse model of cholestasis-induced sarcopenia. We measured mitochondrial mass by TOM20 levels and mitochondrial DNA; ultrastructural alterations by transmission electronic microscopy; mitochondrial biogenesis by PGC-1α plasmid reporter activity and protein levels by western blot analysis; mitophagy by the co-localisation of the MitoTracker and LysoTracker fluorescent probes; mitochondrial potential by detecting the TMRE probe signal; protein levels of OXPHOS complexes and LC3B by western blot analysis; OCR by Seahorse measures; and mtROS by MitoSOX probe signals.

**Results:**

DCA and CA caused a reduction in mitochondrial mass and decreased mitochondrial biogenesis. Interestingly, DCA and CA increased LC3II/LC3I ratio and decreased autophagic flux concordant with raised mitophagosome-like structures. In addition, DCA and CA decreased mitochondrial potential and reduced protein levels in OXPHOS complexes I and II. The results also demonstrated that DCA and CA decreased basal, ATP-linked, FCCP-induced maximal respiration and spare OCR. DCA and CA also reduced the number of cristae. In addition, DCA and CA increased the mtROS. In mice with cholestasis-induced sarcopenia, TOM20, OXPHOS complexes I, II and III, and OCR were diminished. Interestingly, the OCR and OXPHOS complexes were correlated with muscle strength and bile acid levels.

**Conclusion:**

Our results showed that DCA and CA decreased mitochondrial mass, possibly by reducing mitochondrial biogenesis, which affects mitochondrial function, thereby altering potential OCR and mtROS generation. Some mitochondrial alterations were also observed in a mouse model of cholestasis-induced sarcopenia characterised by increased levels of BA, such as DCA and CA.

**Supplementary Information:**

The online version contains supplementary material available at 10.1186/s40659-023-00436-3.

## Introduction

Sarcopenia is characterised by the loss of muscle mass and decreased muscle function [1, 2]. Among the causes of sarcopenia are immobilisation, sepsis, ageing and chronic disease, such as liver disease [2, 3]. We have recently described that bile acids (BA), cholic (CA) and deoxycholic (DCA) are soluble mediators, the levels of which are increased in chronic liver disease. DCA and CA reduce the diameter of C_2_C_12_ myotubes and induce a decrease in myosin heavy-chain protein levels. In addition, the diameters of isolated fibers are reduced by DCA and CA, which are typical features of sarcopenia [4]. This muscle alteration is mediated by the receptor for BA called the TGR5 receptor [4, 5]. Several mechanisms can be involved in muscle alterations, such as decreases in muscle strength and fiber diameter and the diminution of myofibrillar proteins [6, 7], such as unbalanced protein homeostasis, myonuclear apoptosis, oxidative stress, autophagy deregulation and mitochondrial dysfunction [8].

Mitochondria are organelles that are essential for muscle activity. Thus, mitochondria are the primary source of ATP production. They are dependent on oxidative phosphorylation (OXPHOS), which is used in contractile function [3]. The normal function of mitochondria requires OXPHOS components, such as the mitochondrial I, II, III, IV and V complexes [3]. When OXPHOS components have decreased expression or do not function correctly, mitochondrial dysfunction develops, which is characterised by mitochondrial alterations, such as morphological changes, reduced mass and membrane potential and increased mitochondrial reactive oxygen species (mtROS) formation [9, 10].

Mitochondria generate new mitochondria through biogenesis and mitochondrial degradation through mitophagy, a specific autophagy pathway for the elimination of mitochondria [11]. A balance between both processes is essential for maintaining mitochondrial content and cellular metabolism [12, 13]. These processes are closely linked to events of mitochondrial dynamics, such as fusion and fission [14–16]. Mitochondrial biogenesis is led by the peroxisome proliferator-activated receptor gamma coactivator 1-alpha (PGC-1α), a transcriptional coactivator of genes required in several stages of biogenesis, such as Mitochondrial transcription factor A (TFAM) [17]. Generally, when PGC-1α protein levels are increased or post-translationally modified, an increment in mitochondrial biogenesis is observed [18].

Autophagy is a degradative pathway of cellular components, such as proteins and organelles, through a mechanism that involves lysosomes [19]. The autophagic process consists of the formation of double-membrane vesicles called autophagosomes, which are characterised by the presence of microtubule-associated proteins 1A/1B light chain 3B (LC3B) that are post-translationally modified to form LC3II and allow the maturation of autophagosomes. The conversion to an active form of LC3B, represented as the increase in the LC3II/LC3I ratio, is an autophagy activation marker [19, 20]. Mitophagy is a specific autophagy-dependent mitochondrial degradation in which defective mitochondria are degraded to their essential components via lysosomes. Several mitophagy pathways have been described classically as PINK-1/PARKIN, whereas NIX, BNIP3 and FUNDC are alternative stress pathways [21]. The co-localisation of mitochondria with lysosomes has been used as an index of mitophagy.

In the present study, we evaluated the influence of DCA and CA on mitochondrial alterations by detecting mitochondrial mass, potential, bioenergetics, morphology, biogenesis and mitophagy in differentiated C_2_C_12_ cells that modelled skeletal muscle myofibers. In addition, we evaluated mitochondrial alterations in skeletal muscle and isolated muscle fiber in a mouse model of cholestasis-induced sarcopenia characterised by elevated plasma levels of BA.

## Results

### DCA and CA decreased mitochondrial mass and altered mitochondrial structure in C_2_C_12_ myotubes

**Fig. 1:**
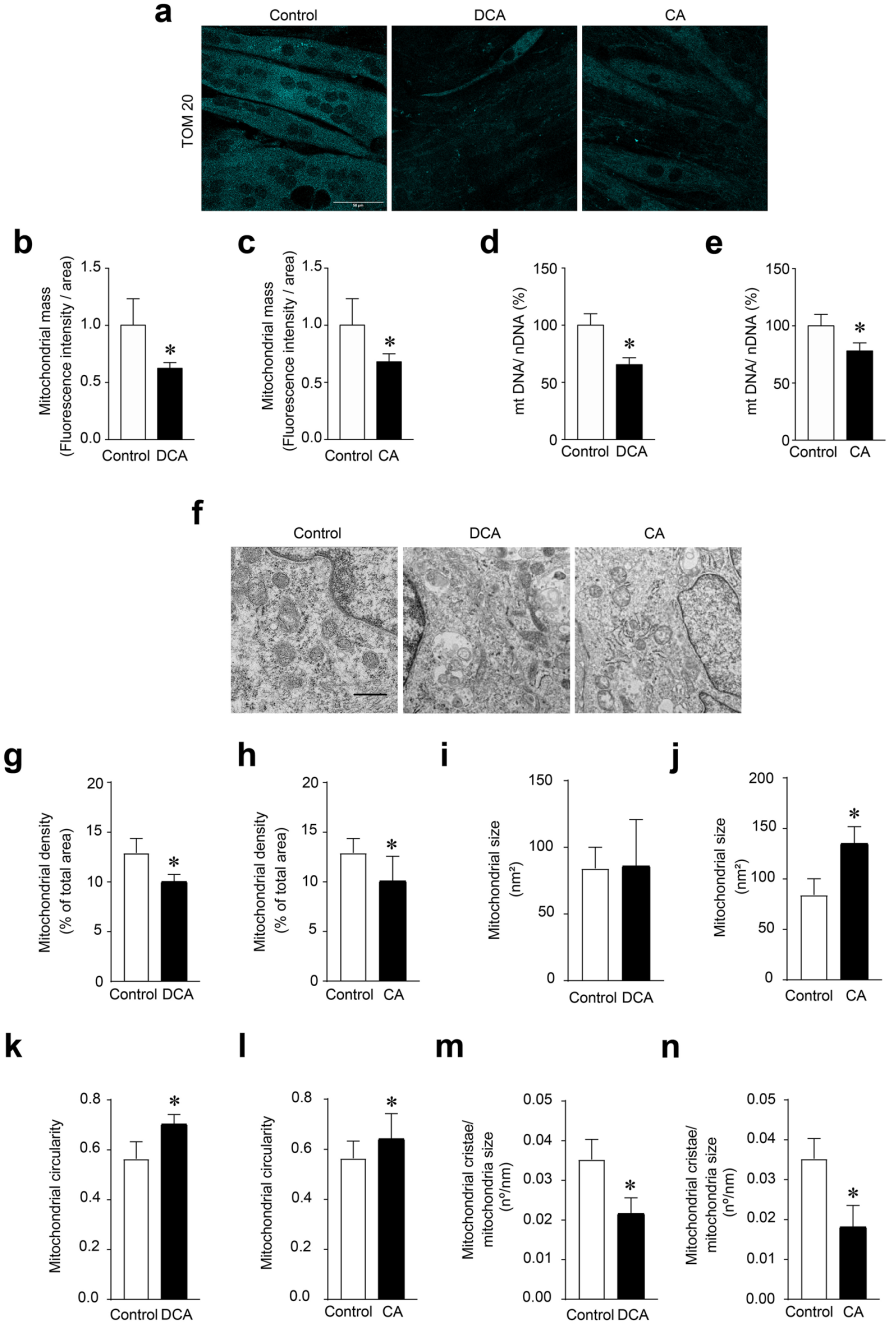
DCA and CA decrease mitochondrial mass in C_2_C_12_ myotubes. Differentiated C_2_C_12_ cells forming myotubes were incubated with 120 μM of DCA or 500 μM of CA for 72 h. When the treatment was finished, indirect immunofluorescence for TOM20 was performed. **a** Representative images for TOM20. Three independent experiments were quantified with four fields of view (areas) analyzed in 25–30 myotubes per condition per each separate experiment. **b**–**c** Analysis of TOM20 immunostaining to quantify mitochondrial mass under DCA (**b**) and CA (**c**) treatment expressed as fold of change. (**d**–**e**) mtDNA and nuclear DNA (nDNA) were determined and expressed as percentages. Quantified mtDNA, normalized by nDNA, in myotubes incubated with DCA (**d**) or CA (**e**). **f** Representative images of mitochondrial from C_2_C_12_ myotubes. Scale bar: 500 μm. For each independent experiment was analyzed the cell mitochondria of at least three fields in four myotubes and were counting 6–8 mitochondria per field. **g**, **h** Determination of mitochondrial density after DCA (**g**) and CA (**h**) treatment was expressed as a percentage of total cellular area. **i**, **j** Mitochondrial size in nm^2^ was calculated in myotubes incubated with DCA (**i**) or CA (**j**). **k**, **l** Mitochondrial circularity was determined as width/lengthy ratio for DCA (**k**) or CA (**l**) treatment. **m**, **n** Finally, the number of mitochondrial cristae was normalized by mitochondria size after incubation with DCA (**m**) and CA (**n**). Values correspond to the mean ± SEM (n = 3 independent experiments for fluorescence microscopy and mtDNA, and n = 4 independent experiments for electron microscopy, *p < 0.05. t-test)

The TOM20 protein is constitutively expressed in the mitochondrial outer membrane, and its abundance is related to the amount of mitochondrial mass [22]. We analysed the effects of DCA and CA on mitochondrial mass by detecting mitochondrial protein TOM20 through indirect immunofluorescence in C_2_C_12_ myotubes. Figure 1a shows representative images of TOM20 immunodetection. The results of the quantitative analysis showed that the fluorescence intensity of TOM20 decreased by 38 ± 5% (Fig. 1b) and 32 ± 7% (Fig. 1c) after the DCA and CA treatments, respectively. Furthermore, we determined mitochondrial DNA (mtDNA) as another parameter of mitochondrial mass. DCA (Fig. 1d) and CA (Fig. 1e) in C_2_C_12_ myotubes decreased the mtDNA content by 35 ± 6% and 22 ± 7%, respectively. In addition, we used a MitoTracker red probe, which consists of a fluorescent dye that enters the mitochondria and is dependent on its potential to target healthy mitochondria (Additional file 1a). The results showed that DCA (Additional file 1b) and CA (Additional file 1c) decreased mitochondrial mass (Control = 1.00 ± 0.15; DCA = 0.60 ± 0.18; CA = 0.69 ± 0.11 fold of change).

To confirm the decreased mitochondrial mass, we analysed the ultrastructure of mitochondria in C_2_C_12_ myotubes treated with DCA and CA using transmission electron microscopy. Figure 1f shows representative images of the experimental groups. As shown in Fig. 1g, h, BA decreased mitochondrial density (Control = 12.90 ± 0.73%; DCA = 10.04 ± 0.35%; CA = 10.10 ± 1.24%) compared with the control group. This result correlated with the decreased mitochondrial mass, as evidenced by the detection of TOM20 (Fig. 1). In addition, only CA increased the mitochondrial size (Control = 84.21 ± 7.9 nm^2^; DCA = 86.20 ± 17.35 nm^2^; CA = 135.20 ± 8.2 nm^2^) (Figs. 1i, j). However, DCA and CA increased mitochondrial circularity (i.e., width and length), which affected mitochondrial structure (0.70 ± 0.02 and 0.64 ± 0.04 of DCA and CA, respectively), compared with 0.56 ± 0.03 in the control group (Fig. 1k, l). In addition, both BA decreased the mitochondrial cristae number (Control = 0.035 ± 0.003 cristae/nm; DCA = 0.022 ± 0.002 cristae/nm; CA = 0.018 ± 0.003 nm^2^) (Fig. 1m, n). These results indicate that structural alterations could also be conducted to impair mitochondrial activity. Thus, we confirmed alterations in mitochondrial structure, which suggested that mitochondrial machinery could be dysfunctional.

Mitochondria can exhibit either homogenous or punctate morphological patterns, which may indicate changes in their function [23]. The MitoTracker red probe allows for the visualisation of homogeneous or punctate patterns. We used the MitoTracker red probe in C_2_C_12_ myotubes treated with DCA and CA to assess the mitochondrial distribution pattern. Additional file 1a shows that the myotubes presented a homogeneous signal under control conditions, whereas DCA and CA induced a punctate pattern. The quantification indicated differences in mitochondrial distribution because there was a more significant number of myotubes with a punctuate pattern in the DCA and CA conditions than in the control condition. DCA increments were 4.3 ± 0.6-fold (Additional file 1d), while CA increments were 3.5 ± 0.7-fold (Additional file 1e). These results demonstrated that DCA and CA decreased mitochondrial mass and altered the ultrastructure and distribution patterns of mitochondria.

### DCA and CA decreased mitochondrial biogenesis and autophagic flux in C_2_C_12_ myotubes

**Fig. 2:**
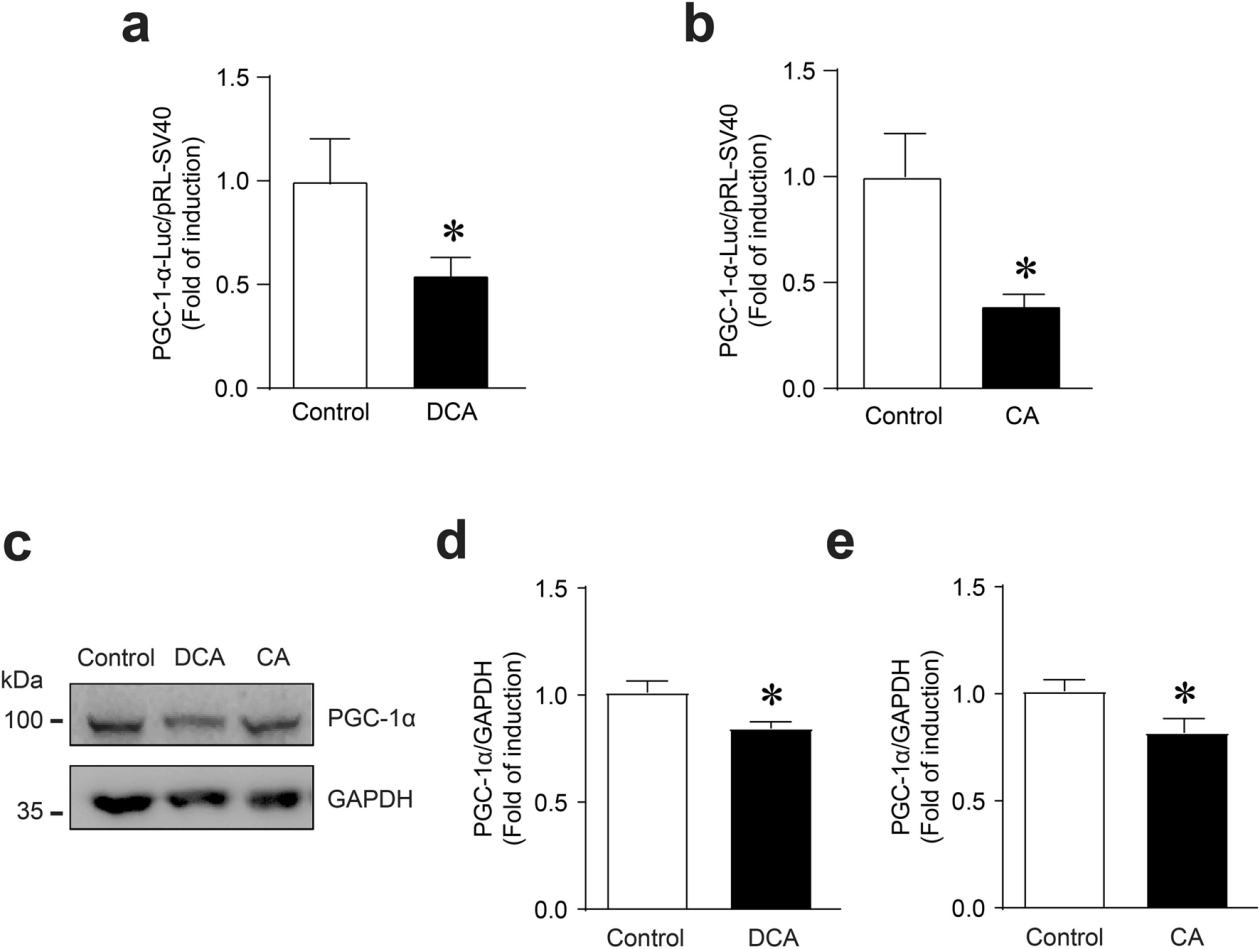
DCA and CA decrease mitochondrial biogenesis in C_2_C_12_ myotubes. **a**–**b** C_2_C_12_ cells were co-transfected with the plasmid containing PGC-1α promoter 2 kb coupled to luciferase gene and pRL-SV40 using Lipofectamine 3000. Further, the cells were differentiated for 4 days, and myotubes were incubated with 120 μM of DCA (**a**) and 500 μM of CA (**b**) for 24 h. Dual luciferase activities were measured and expressed as a fold of change. Values correspond to the mean ± SEM (n = 3 independent experiments, *p < 0.05. t-test. **c**–**e** C_2_C_12_ myotubes were incubated with 120 μM of DCA, and 500 μM of CA for 72 h, and protein levels of PGC-1α were detected by Western blot analysis using GAPDH levels as the loading control. Molecular weight markers are depicted in kDa. The quantitative analysis of value is expressed as folds of change for DCA (**d**) and CA (**e**). Values correspond to the mean ± SEM (n = 3 independent experiments, *p < 0.05. t-test)

The PGC-1α transcriptional coactivator has been described as the master regulator of mitochondrial biogenesis [24]. To evaluate changes in mitochondrial biogenesis that could explain the decrease in mitochondrial mass, we analysed the effects of DCA and CA on PGC-1α levels in C_2_C_12_ myotubes. First, we determined the effects of these BA on a plasmid reporter containing part of the PGC-1α promoter. The results showed that the DCA (Fig. 2a) and CA (Fig. 2b) treatments decreased PGC-1α plasmid reporter activity. These results were concordant with PGC-1α protein levels evaluated by western blot analysis (Fig. 2c). The quantitative analysis showed that DCA (Fig. 2d) and CA (Fig. 2e) decreased PGC-1α protein levels (Control = 1.00 ± 0.05; DCA = 0.85 ± 0.03; CA = 0.82 ± 0.06-fold change). Both analyses showed that DCA and CA diminished the expression and protein levels of PGC-1α, suggesting altered mitochondrial biogenesis.

**Fig. 3:**
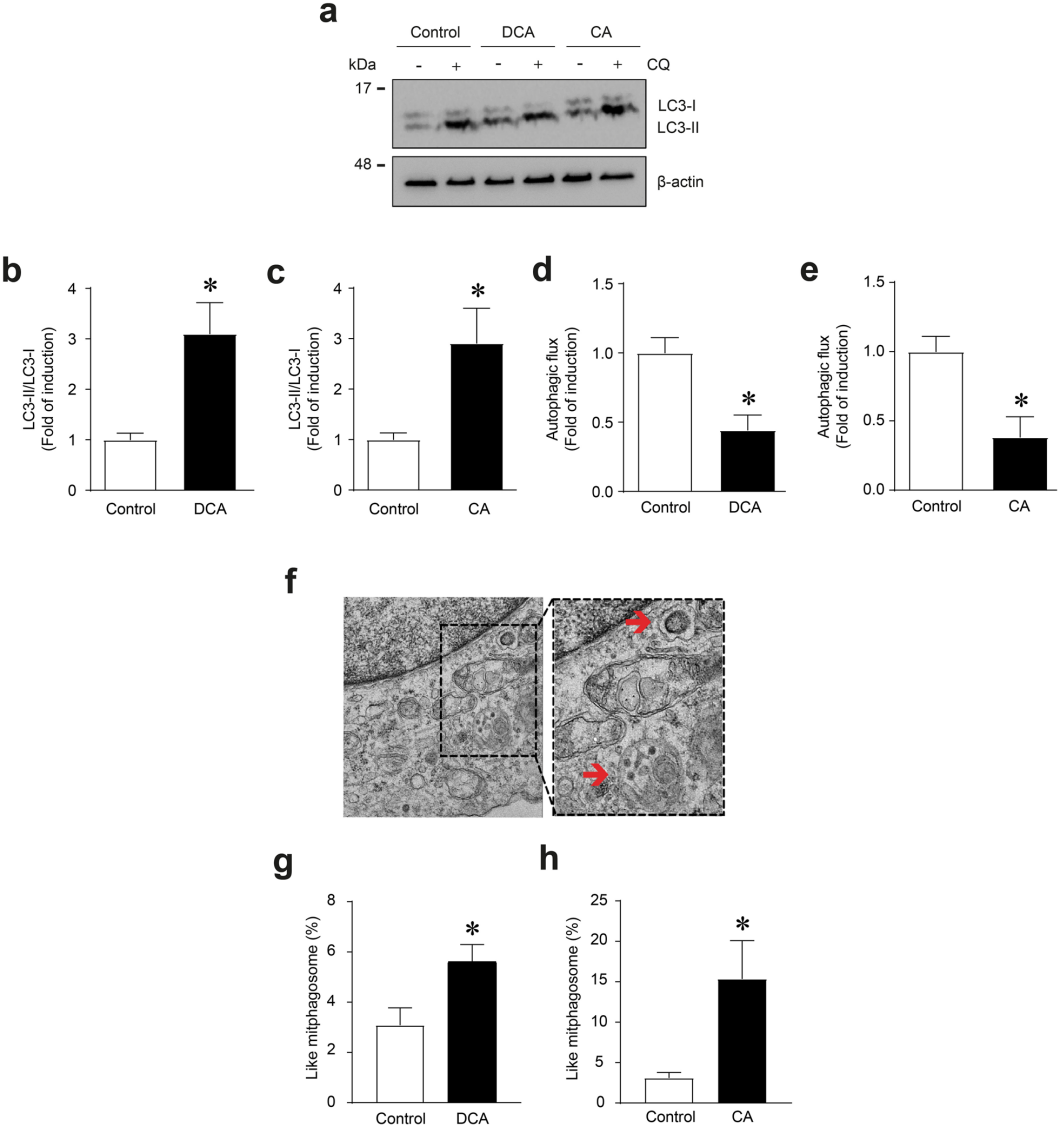
DCA and CA decrease autophagic flux in C_2_C_12_ myotubes. C_2_C_12_ myotubes were incubated with 120 μM of DCA or 500 μM of CA for 72 h. To block the autophagy process, 8 h before finishing the experiment, the myotubes were co-treated with chloroquine (CQ; 50 μM). **a** When the treatment was completed, we evaluated LC3I and LC3II protein levels through Western blot. β-actin levels are shown as the loading control. Molecular weight markers are depicted in kDa. **b**–**c** The results were represented as the LC3II/LC3I ratio and expressed as the mean ± SEM (fold of change) in myotubes incubated with DCA (**b**) and CA (**c**). **d–e** The quantitative analysis of autophagic flux was conducted using densitometric analysis of LC3II levels obtained by comparing the difference between the condition with and without CQ from the representative images shown in (a). **f** Representative pictures of mitophagosome-like structures from C_2_C_12_ myotubes. Scale bar: 500 μm. For each independent experiment was analyzed the cell mitochondria of at least three fields in four myotubes and were counting 6–8 mitochondria per field. **g**, **h** The percentage of mitophagosome-like structures observed in myotubes after DCA (**g**) or CA (**h**) treatment was calculated. The values were expressed as the mean ± SEM (n = 3 independent experiments for immunoblotting, and = 4 independent experiments for electron microscopy, *p < 0.05. t-test)

Autophagy is a process involved in the clearance of dysfunctional mitochondria (i.e., mitophagy) by the formation of autophagosomes. Conversion from the cytoplasmic form of the LC3B protein (LC3I) to the autophagosome-incorporated form (LC3II) serves as a marker of autophagosome formation [19, 21]. Therefore, we evaluated the effects of DCA and CA on autophagy signaling by detecting the LC3II/LC3I ratio using western blot analysis (Fig. 3a). In addition, chloroquine treatment blocks fusion between autophagosomes and lysosomes by altering the acidic environment of lysosomes and allowing the determination of autophagy flux. Hence, we evaluated the effects of DCA and CA on autophagic flux using chloroquine as a lysosome inhibitor (Fig. 3a). The results showed that DCA (Fig. 3b) and CA (Fig. 3c) increased the LC3II/LC3I ratio by 3.10 ± 0.62 and 2.19 ± 0.61-fold inductions, respectively. The comparison of differences among LC3II protein levels showed that DCA (Fig. 3d) and CA (Fig. 3e) decreased the autophagic flux to 0.44 ± 0.11 and 0.38 ± 0.19-fold, respectively. We used electron microscopy to determine the influence of DCA and CA on the percentage of mitophagosome-like structure accumulation in C_2_C_12_ myotubes to confirm the decrease in autophagic flux. Figure 3f shows a representative mitophagosome-like structure. We observed an increase in mitophagosome percentages of DCA (5.61 ± 0.68%) (Fig. 3g) and CA (15.35 ± 4.73%) (Fig. 3h) compared with the control condition (3.08 ± 0.69%).

Mitochondrial mass is determined by a balance between biosynthesis and degradation processes characterised by the participation of different proteins. PINK-1 is a protein associated with mitophagy. Thus, when synthesised, PINK-1 targets mitochondria and is quickly internalised because of the mitochondrial membrane potential and then degraded. However, when mitochondria are damaged, they lose their potential, and the importation of PINK-1 fails, accumulating in the outer membrane of the mitochondria and triggering a series of signals interpreted as damage and favouring recognition by autophagosomes. Therefore, the accumulation of PINK-1 indicates an increase in mitophagy [21]. Hence, we evaluated the effects of DCA and CA on mitophagy in C_2_C_12_ myotubes by detecting PINK-1 levels as critical markers in the machinery involved in mitophagy (Additional file 2a). The results showed that DCA (Additional file 2b) and CA (Additional file 2c) decreased PINK-1 protein levels (Control = 1.00 ± 0.06; DCA 0.61 ± 0.15; CA = 0.67 ± 0.11).

**Fig. 4:**
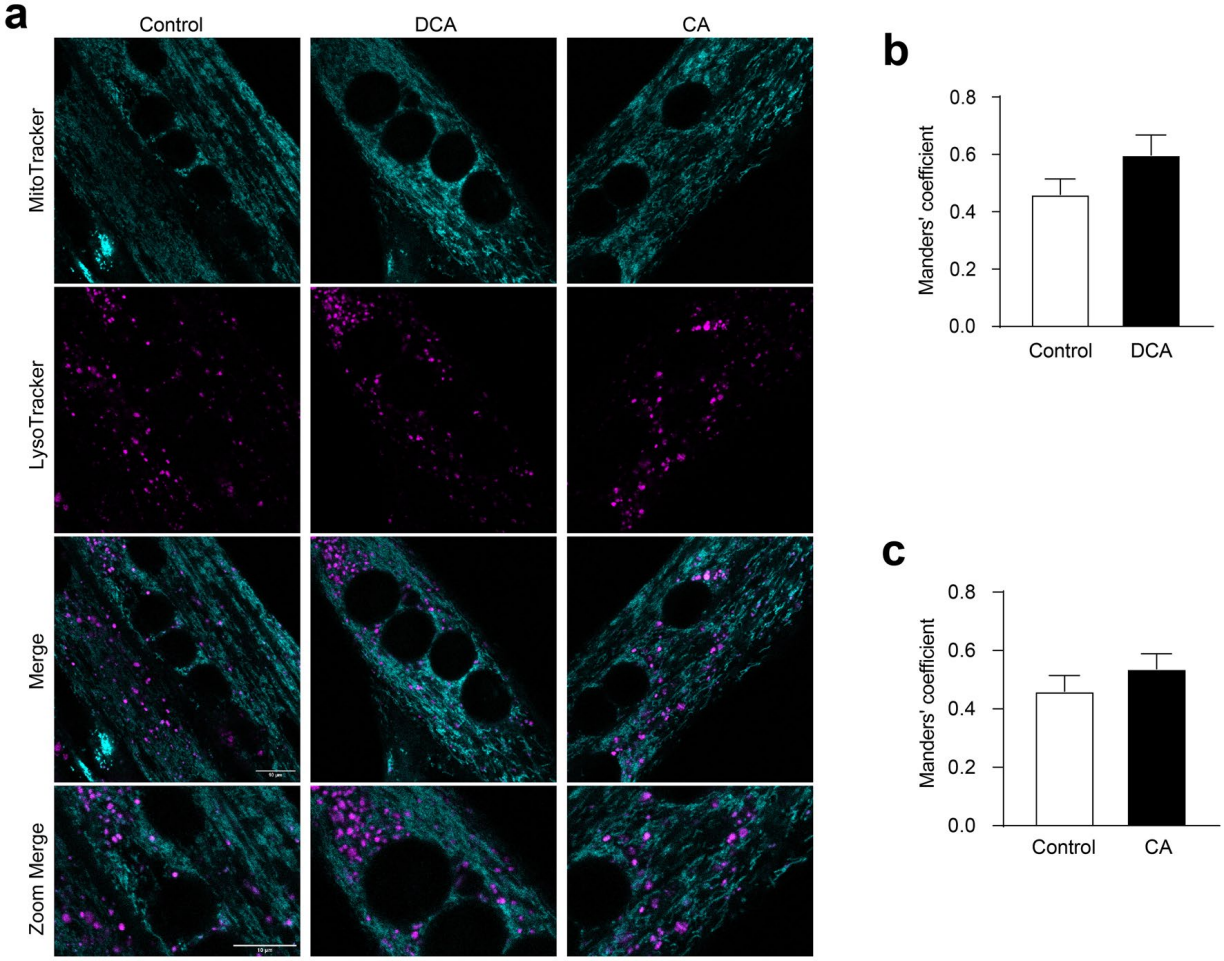
Mitophagy is unchanged by DCA and CA in C_2_C_12_ myotubes. **a**–**c** C_2_C_12_ myoblasts were differentiated for 5 days until forming myotubes. Then, myotubes were incubated with 120 μM of DCA or 500 μM of CA for 48 h. **a** After treatment with BA, cell culture was co-incubated with Mitotracker green FM (first panel) and Lysotracker Red DND-99 (second panel) probes. The co-localization (third panel and forth panel) between Mitotracker green and Lysotracker signals was determined as a parameter of mitophagy. Scale bar: 10 μm. Co-localization degree of fluorescent signals was calculated by Manders' coefficient using the JACoP plugin by ImageJ for (**b**) DCA and (**c**) CA. The values indicate the mean ± SEM of independent random fields. Scale bar:10 μm. (n = 3 independent experiments, *p < 0.05. t-test)

When the autophagosomes capture damaged mitochondria, they fuse with lysosomes to form autolysosomes, a structure in which the degradation of this organelle occurs [25]. Therefore, an increase of mitochondria in lysosomes indicates an increase in their degradation. To determine whether DCA and CA treatments affected mitochondria degradation, we determined the co-localisation of mitochondria (labelled with MitoTracker green) with lysosomes (tagged with lysotracker) (Fig. 4a). The Manders’ coefficient indicated that DCA (Fig. 4b) and CA (Fig. 4c) did not alter the mitochondria or lysosome co-localisation. These results showed that DCA and CA did not increase mitophagy in the C_2_C_12_ cells. These results indicate that diminished mitochondrial mass induced by DCA and CA may be associated with biogenesis impairment and decreased autophagy flux but not with increasing mitochondrial degradation.

### DCA and CA decreased the mitochondrial potential, oxygen consumption rate, and OXPHOS components in C_2_C_12_ myotubes

**Fig. 5:**
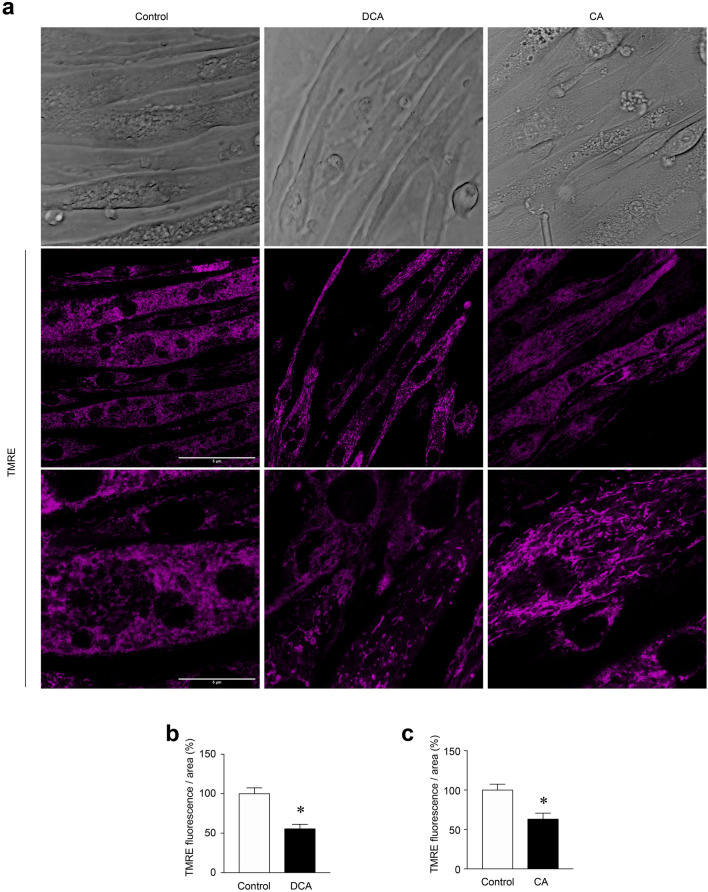
The mitochondrial potential is decreased by DCA and CA in C_2_C_12_ myotubes. (**a**–**b**) Myotubes were incubated with 120 μM of DCA or 500 μM of CA for 72 h. Then, cells were incubated with a TMRE probe for 30 min. **a** Representative images for TMRE signal. Phase contrast (upper panel), TMRE (middle panel), and zoom of TMRE signal (lower panel). Scale bar: 5 μm. **b**–**c** Analysis for TMRE fluorescence in myotubes incubated with DCA (**b**) and CA (**c**). Three independent experiments were quantified with four fields of view (areas) analyzed in 25–30 myotubes per condition per each separate experiment. Values are expressed as percentages and correspond to the mean ± SEM (n = 3 independent experiments, *p < 0.05. t-test)

The mitochondrial membrane potential generated by complexes I, III and IV is essential for energy generation [3]. In addition, the mitochondrial membrane potential plays a crucial role in mitochondrial homeostasis because it enables the import of proteins and ions that are essential for the functioning of the mitochondria [26]. Therefore, changes in mitochondrial membrane potential may suggest mitochondrial dysfunction. TMRE is a cationic probe that rapidly enters functional mitochondria due to its negative charge. However, depolarised or inactive mitochondria that lose their membrane potential fail to sequester this probe. We determined the influence of DCA and CA on mitochondrial potential using the TMRE probe. Figure 5a shows C_2_C_12_ myotubes (upper panel) treated with DCA and CA, TMRE signal (middle panel), and zoom (lower panel). The results of the treatment with DCA showed a decreased TMRE signal (Control = 100 ± 12.8; DCA = 44.4 ± 5.7%) (Fig. 5b) and CA (Control = 100 ± 12.8; CA = 37 ± 7.7%) (Fig. 5c).

**Fig. 6:**
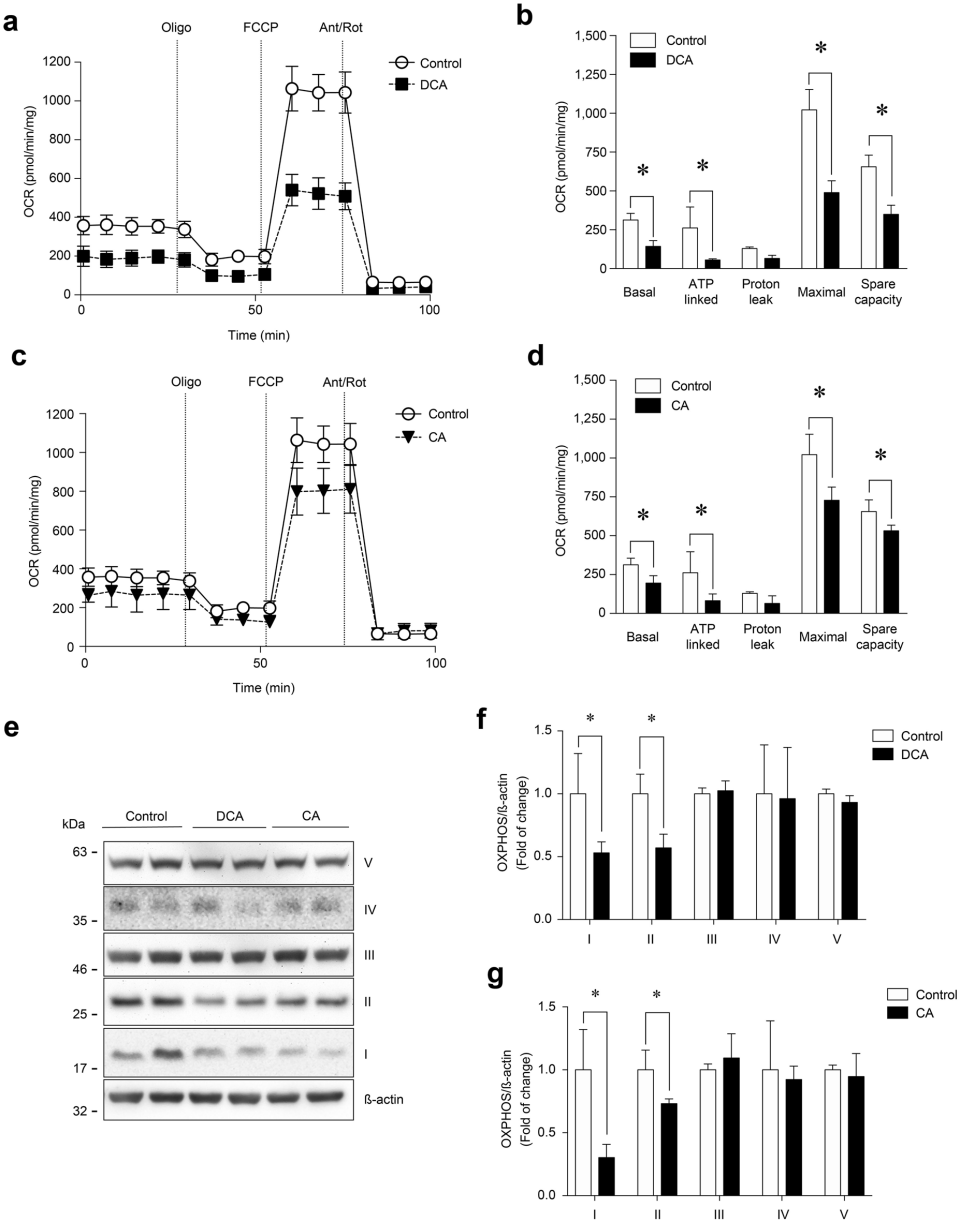
DCA and CA decrease the OCR and OXPHOS complexes I and II in C_2_C_12_ myotubes. Differentiated C_2_C_12_ cells forming myotubes were incubated with 120 μM of DCA (**a**–**b**) or 500 μM of CA (c-d) for 72 h. Basal, ATP-linked, H^+^-leak, maximal and spare OCR were determined as described in the Materials and Methods section. Values are expressed as pmol/min/mg and correspond to the mean ± SEM (n = 3, *p < 0.05. T-test). **e** Protein levels of I, II, III, and IV mitochondrial OXPHOS complexes were detected by Western blot analysis using β-actin levels as the loading control. Molecular weight markers are depicted in kDa. The quantitative value analysis is expressed as a fold of change for DCA (**f**) and CA (**g**). Values correspond to the mean ± SEM (n = 3 independent experiments, *p < 0.05. t-test)

To confirm whether alterations in mitochondrial membrane potential affected its functionality in response to DCA and CA, we measured the OCR in C_2_C_12_ myotubes using the XF24-3 Seahorse Flux Analyzer, which is a key indicator of mitochondrial oxygen consumption [27]. DCA treatment (Fig. 6a) decreased basal OCR (control = 312.7 ± 43.0 vs DCA = 143.2 ± 37.3 pmol/min/mg). Next, oligomycin, a V complex inhibitor, was added to determine ATP-linked OCR by subtracting the oligomycin rate from the baseline cellular OCR. The DCA treatment decreased ATP-linked OCR (control = 261.7 ± 81.6 vs DCA = 55.3 ± 7.3 pmol/min/mg). FCCP was then added to collapse the inner membrane gradient, allowing OXPHOS to work at the maximal rate and the mitochondria to reach their maximal respiratory capacity by subtracting non-mitochondrial respiration from the FFCP rate. DCA reduced maximal respiration induced by FCCP (control = 1,021.4 ± 131.5 vs DCA = 489.6 ± 75.9 pmol/min/mg). Mitochondrial spare OCR was calculated by subtracting basal respiration from maximal respiratory capacity. The DCA treatment also reduced spare OCR (control = 655.0 ± 76.2 vs DCA = 349.2 ± 58.6 pmol/min/mg) (Fig. 6b). As shown in Fig. 6b, DCA treatment did not change oligomycin-insensitive OCR or proton leakage (subtracting non-mitochondrial respiration from the ATP-linked OCR by oligomycin rate). Figure 6c shows similar results of OCR when the C_2_C_12_ myotubes were incubated with CA (basal OCR: control = 312.7 ± 43.0 vs. CA = 195.7 ± 47.9 pmol/min/mg; ATP-linked OCR: control = 261.7 ± 81.6 vs. CA = 81.6 ± 44.4 pmol/min/mg; FCCP-induced maximal respiration: control = 1021.4 ± 131.5 vs. CA = 728.1 ± 84.6 pmol/min/mg; spare OCR: control = 655.0 ± 76.2 vs. CA = 532.4 ± 36.2 pmol/min/mg) (Fig. 6d). Similar to the results for DCA, H^+^-leak OCR was unchanged by the CA treatment (Fig. 6d).

One explanation for the loss of mitochondrial potential and functionality was the alteration of the OXPHOS complexes. Therefore, we evaluated the effects of DCA and CA on the protein levels of OXPHOS complexes using western blot analysis (Fig. 6e). The results showed that DCA (Fig. 6f) and CA (Fig. 6g) decreased OXPHOS complex I (Control = 1.00 ± 0.28; DCA = 0.54 ± 0.08; CA = 0.30 ± 0.11-fold of change) and complex II (Control = 1.00 ± 0.12; DCA = 0.58 ± 0.09; CA = 0.73 ± 0.03-fold change). The same figures show that in complexes III and V, the DCA and CA treatments did not modify the protein levels.

These results showed that DCA and CA decreased mitochondrial function by altering mitochondrial-dependent OCR and OXPHOS complexes I and II in C_2_C_12_ myotubes.

### DCA and CA induce mitochondrial ROS (mtROS) increments in C_2_C_12_ myotubes

**Fig. 7:**
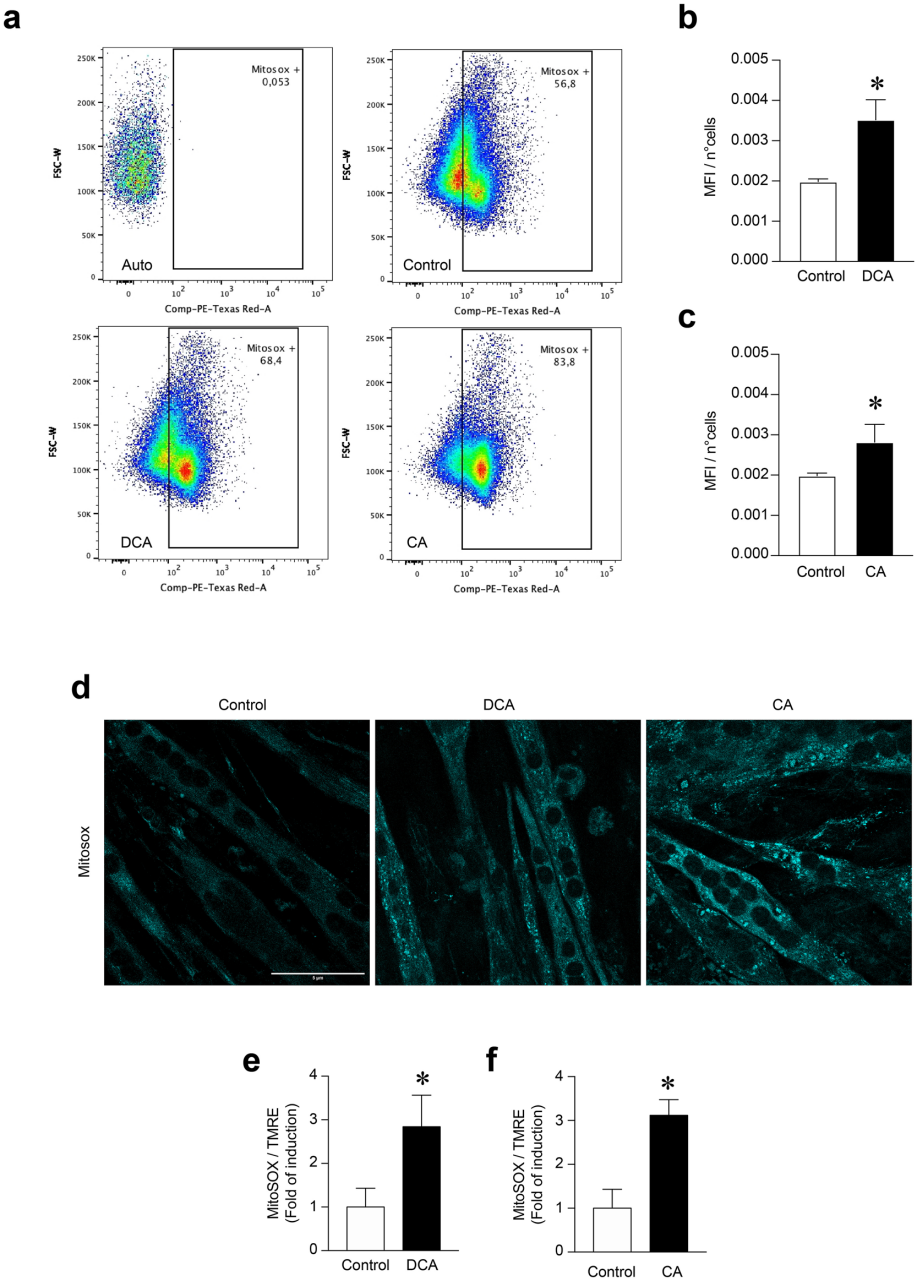
Increased mitochondrial ROS levels induced by DCA and CA in C_2_C_12_ myotubes. C_2_C_12_ myoblasts were differentiated for 5 days until forming myotubes. Then, cells were incubated with 120 μM of DCA or 500 μM of CA for 72 h. Further, myotubes were incubated with 10 μM or 5 μM of MitoSOX probe for flow cytometry and fluorescence microscopy analysis, respectively. **a** Representative flow cytometry dot plot analyses to identify mean fluorescence intensity (MFI) for each condition. **b**–**c** Analysis of the MFI normalized by the number of cells (n°cells) in myotubes incubated with DCA (**b**) or CA (**c**). Values are expressed as the mean of MFI/n°cells ± SEM (n = 3 independent experiments, *p < 0, 05. t-test). (**d**) Representative images for the MitoSOX signal. , **f**) Analysis of the MitoSOX fluorescence normalized by TMRE signal in myotubes incubated with DCA (**e**) or CA (**f**). Scale bar: 5 μm. Values are expressed as a fold of induction and correspond to the mean ± SEM (n = 3 independent experiments, *p < 0.05. t-test)

Mitochondrial dysfunction increases the ROS levels produced by this organelle, contributing to exacerbated functional alterations [28]. In this context, the MitoSOX Red probe corresponded to a stain incorporated into mitochondria and, in response to oxidation, fluoresces and indicates mtROS levels. To assess whether DCA and CA could increase mtROS, we used flow cytometry to evaluate whether they affected mtROS levels by detecting the MitoSOX fluorescent signal in C_2_C_12_ myotubes. Figure 7a shows a dot plot of the MitoSOX fluorescent signal, which increased with the DCA and CA treatments. Specifically, the MitoSOX fluorescent signal increased 1.77-fold in DCA (Fig. 7b) (DCA: 0.00519 ± 0.00029 vs Ctrl: 0.001985 ± 0.000381) and 1.42-fold in CA (Fig. 7c) with respect to the Control (CA: 0.002820 ± 0.000438 vs Ctrl: 0.001985 ± 0.000381).

Furthermore, MitoSOX-associated mtROS were evaluated using fluorescence microscopy. Figure 7d shows that the MitoSOX signal increased in the DCA and CA treatments. The results showed that the MitoSOX-associated mtROS increment induced by DCA (Fig. 7e) and CA (Fig. 7f) was 2.8 ± 0.9- and 3.1 ± 0.4-fold of induction, respectively. These results indicate that DCA and CA increase mtROS in C_2_C_12_ myotubes.

### Mitochondrial alterations correlate with strength and bile acids in mice with cholestasis-induced sarcopenia

**Fig. 8:**
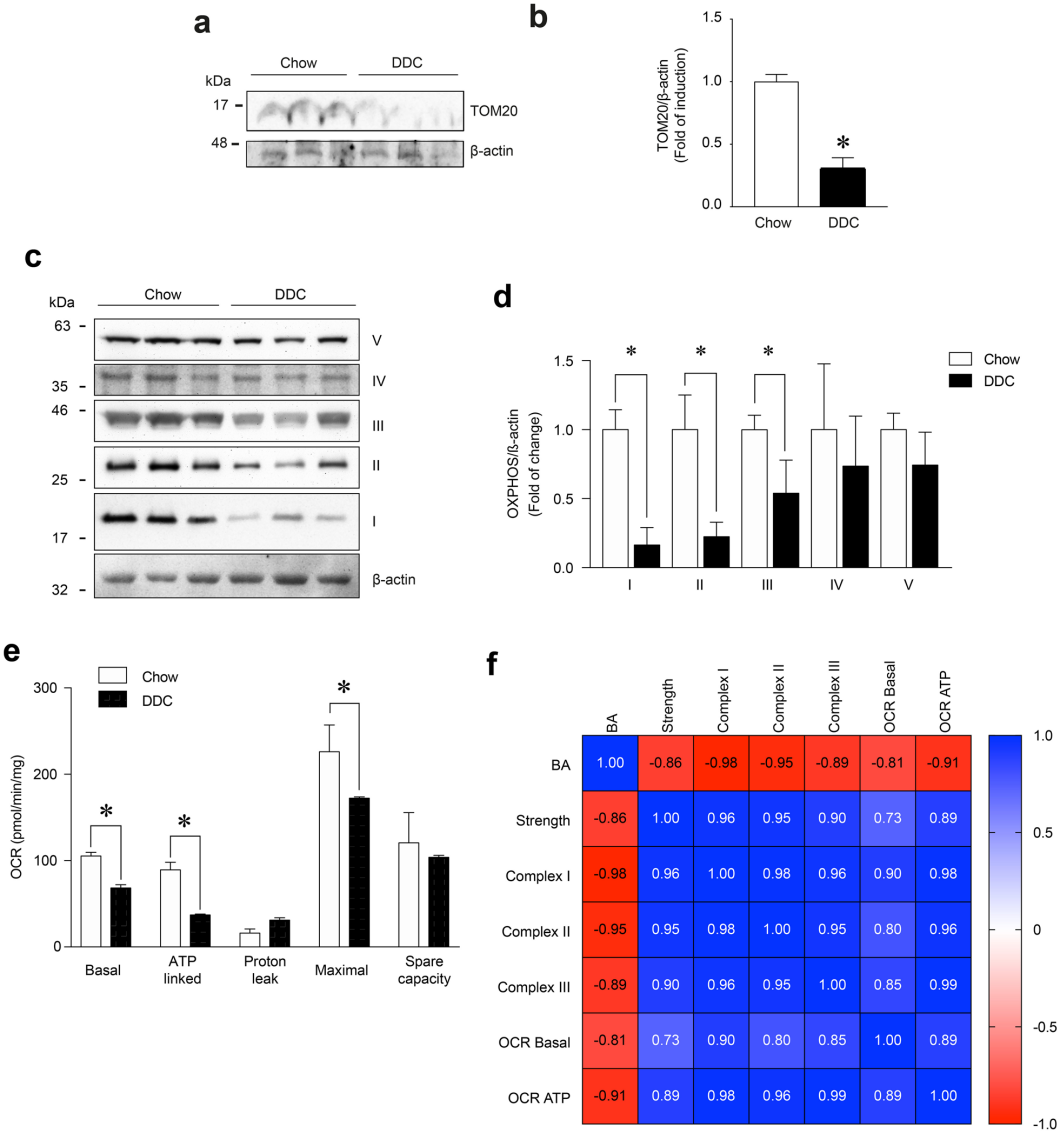
Muscle strength and bile acids correlate with mitochondrial alterations in a mouse model of cholestasis-induced sarcopenia. C57BL/6 mice were fed with a chow or a DDC-supplemented diet for 6 weeks. After the treatment, the TA muscles were excised and homogenized to evaluate (**a**) TOM20 and (**c**) I, II, III, and IV mitochondrial OXPHOS complexes protein levels by Western blot using β-actin levels as the loading control. Molecular weight markers are depicted in kDa. The quantitative value analysis is expressed as a fold of change for TOM20 (**b**) and OXPHOS complexes (**d**) compared to chow diet animals. **e** The basal, ATP-linked, H^+^-leak, maximal, and spare OCR of isolated EDL muscle fibers was evaluated with seahorse XF Cell Mito Stress as described in the Materials and Methods section. The values correspond to the mean ± SEM (n = 5–7 animals per condition, *p < 0.05 vs chow diet. t-test). **f** Heatmap of Pearson correlation for bile acids levels, strength, OXPHOS complexes, ORC basal, and ORC ATP represent the relation among different variables

We previously described a mouse model of cholestasis-induced sarcopenia induced by hepatotoxin (DDC) intake [29]. This model is characterised by decreased muscle strength (Additional file 3a) and increased plasma levels of BA, including DCA and CA (Additional file 3b) [29]. These parameters showed an inverse correlation (Pearson coefficient = − 0.86; p < 0.001) (Additional file 3c). In sarcopenia, fast-twitch muscles are the primary muscles affected compared with slow-twitch muscles. Thus, we utilised two fast muscles, *Tibialis anterior* (TA) and *Extensor digitorum longus* (EDL), to determine whether mitochondrial parameters were altered in this model [30–32]. To describe the mitochondrial mass and function and correlate them with muscle function and BA levels, we applied western blot analysis to measure TOM20 and OXPHOX complex protein levels in skeletal muscle in mice fed a standard (Chow) or DDC-supplemented diet (DDC). As shown in Figs. 8a, b, TOM20 levels (DDC = 0.306 ± 0.086-fold of change) decreased in TA muscle from DDC-fed mice compared with the chow group (Chow = 1.000 ± 0.0579-fold of change). This result indicated that mitochondrial mass decreased in the sarcopenic muscles of the cholestatic mice.

We then applied western blot analysis to evaluate the effects of DDC diet-induced cholestasis on the protein levels of the OXPHOS complexes (Fig. 8c). The results (Fig. 8d) indicated that DDC decreased OXPHOS complexes I, II and III by 83.5 ± 12.5, 77.6 ± 10.4, and 46.1 ± 24%, respectively, compared with the TA muscle chow group. The same figures show that the protein levels in complexes IV and V were unmodified under the DDC diet.

To evaluate whether alterations in mitochondrial mass and OXPHOS complexes affected mitochondrial function, we determined OCR in EDL muscle fibers isolated from mice fed chow and DDC diets. Figure 8e shows that the DDC diet decreased basal OCR (chow = 105.5 ± 4.1 vs DDC = 68.4 ± 3.6 pmol/min/mg), ATP-linked OCR (chow = 89.4 ± 8.8 vs DDC = 37.2 ± 1.0 pmol/min/mg), and the maximal respiration induced by FCCP (chow = 226.1 ± 31 vs DDC = 172.4 ± 1.5 pmol/min/mg) in EDL muscle fibers.

We also evaluated potential correlations between muscle strength, BA levels, OCR and OXPHOS complexes. As shown in Fig. 8f, a positive correlation was found between muscle strength and OCR basal (Pearson coefficient = 0.73; p < 0.05), ATP-linked OCR (Pearson coefficient = 0.89; p < 0.001), and OXPHOS complexes I (Pearson coefficient = 0.96; p < 0.01), II (Pearson coefficient = 0.95; p < 0.05) and III (Pearson coefficient = 0.90; p < 0.05). Figure 8f also shows that an inverse correlation was found between BA levels and OCR basal (Pearson coefficient = -0.81; p < 0.05), ATP-linked OCR (Pearson coefficient = -0.91; p < 0.001), and OXPHOS complexes I (Pearson coefficient = -0.98; p < 0.001), II (Pearson coefficient = − 0.95; p < 0.05) and III (Pearson coefficient = − 0.89; p < 0.05).

## Discussion

In the present study, we evaluated the effects of two BA—DCA and CA—on mitochondrial mass and function, biogenesis, mitophagy, autophagic flux and ultrastructure. The results showed that DCA and CA decreased mitochondrial mass, which was determined by TOM20 levels, MitoTracker red intensity, mtDNA content, and mitochondrial density. The results also showed decreased function, measured by reduced OCR and potential, as well as increased mtROS levels and ultrastructural alterations, measured by increased circularity and decreased cristae numbers. In addition, the results showed that DCA and CA decreased the autophagic flux concomitantly with the accumulation of mitophagosome-like structures. The results also indicated that DCA and CA decreased mitochondrial biogenesis but did not provide evidence of changes in mitophagy. Interestingly, the mice with cholestasis-induced sarcopenia had decreased TOM20, OXPHOS complexes I, II and III, and OCR. In addition, the OCR and OXPHOS complexes were correlated with muscle strength and BA levels.

The decrease in mitochondrial mass caused by DCA and CA incubation can be explained by changes in mitochondrial biogenesis induced by these BA. The differences observed in PGC-1α protein levels support this finding. Moreover, the decrease in PGC-1 α promotor activity suggests that the participation of regulatory mechanisms at the transcriptional level is induced by DCA and CA, which affects mitochondrial biogenesis. However, our results contradict those of previous studies in the literature. In endothelial cells, taurolithocholic acid (TLCA) increased PGC-1α levels and activity, which translated into an improved mitochondrial function [33]. INT677, an agonist of farnesoid and TGR5, both receptors for BA, induced mitochondrial biogenesis in fat tissue [34]. In addition, ursodeoxycholic acid (UDCA) promoted mitochondrial biogenesis in the livers of obese mice [35]. However, our results showed impaired mitochondrial biogenesis, possibly due to a tissue-specific mechanism in skeletal muscle. In this context, we must consider that skeletal muscle has two populations of mitochondria, subsarcolemmal (SS) and intermyofibrillar (IMF), which differ according to their subcellular localisation, morphology and biochemical properties [36–38]. The existence of organellar heterogeneity has enabled determining the differential responses of IMF and SS to conditions such as the regulation of fatty acid metabolism or under conditions of disuse or exercise [39–41]. The differential responses of the two mitochondrial subfractions and the differential import of mitochondrial proteins to these fractions [42] imply a potential heterogeneous regulation of mitochondrial biogenesis influenced by nuclear domains or extracellular signals, such as BA [43]. In this context, it has been described that the cell response to high BA levels induces cell death by apoptosis, a process dependent on mitochondria, in hepatocytes [44]. However, skeletal muscle in a cholestatic condition with high BA levels shows increases in some markers of apoptosis without producing the death of muscle fiber [45]. This event could be explained by the differential responses of IMF and SS mitochondria in muscles exposed to BA.

Another explanation for the discrepancy in our results between mitochondrial biogenesis in muscle cells and other cell types is the metabolic context in which BA can act. Regarding endothelial cells in which BA increased mitochondrial biogenesis, experiments were conducted in obese and diabetic contexts, and the results showed that BA had beneficial effects. It has been demonstrated that BA, administered via the intestine, indirectly improved insulin resistance through the secretion of GLP-1 and insulin, with the participation of gut microbiota [46]. In the present study, the mice were not obese or diabetic. The increased BA levels in plasma generated sarcopenia in the skeletal muscle [4, 29], altering the mass and function of mitochondria and showing decreased mitochondrial biogenesis.

Combined with mitochondrial biogenesis, mitophagy establishes a balance that preserves mitochondrial homeostasis. Interestingly, the decreased mitochondrial mass induced by DCA and CA did not involve changes in mitophagy. The evidence is sufficient to link biogenesis, mitophagy and the balance between them. The mTOR signaling pathway is typically involved in this relationship [47]. Another mediator engaged in sensing energy expenditure with consequences for mitochondrial biogenesis, and mitophagy is AMPK [48]. Previous results have shown that mitochondrial dynamics, such as fission, are linked with biogenesis and mitophagy. This relationship has been described as a mechanism that involves a distinctive signature of fission (peripherical or middle) associated with inducing biogenesis or mitophagy [49].

The changes observed in mitochondrial mass can be translated into functionality. Our results showed that DCA and CA generated mitochondrial dysfunction. Antecedents in aged muscle cells indicate that the reduction in mitochondrial function could be caused by a decrease in mitochondrial volume, an increase in oxidative stress, an accumulation of mutations in mtDNA, and/or altered mitochondrial morphology [50]]. Our results showed that DCA and CA produced decreased potential and OCR, which are indicative of mitochondrial dysfunction. These features are also related to changes in the OXPHOS components, such as I and II complexes. Similarly, we found that DCA and CA increased circularity and decreased the cristae number, two parameters associated with mitochondrial malfunction. We observed increased size and circularity, which could indicate mitochondrial fusion and the presence of damaged mitochondria. Mitochondria form a highly dynamic and interconnected network in skeletal muscle [51]. Our results showed a possible disruption of the mitochondrial network in the presence of DCA and CA, changing from a homogeneous network to a punctate pattern. Thus, these findings suggest that both BAs produce decreased mitochondrial mass and dysfunction of the mitochondrial pool, which are probably associated with ATP production and mtROS.

We previously demonstrated that DCA and CA induced oxidative damage in myotubes and muscle fibers [4]. In the present study, we found that DCA and CA increased mtROS levels, which likely contributed to the development of oxidative stress in muscle cells. In this context, the depletion of antioxidant mechanisms could also be associated with increased oxidative stress in skeletal muscle cells treated with DCA and CA. DCA diminishes mRNA and levels of antioxidant mitochondrial protein peroxiredoxin in trophoblast cells. The knockdown of this family of peroxidase enzymes resembles the oxidative stress and mitochondrial dysfunction induced by BA [52]. In addition, mitochondrial alterations in bioenergetics activity and ETC could be associated with the alteration of electron transport within this organelle, consequently favouring the accumulation of mtROS and causing elevated mitochondrial oxidative stress. However, further analysis must be conducted to determine the reason for the increased mtROS induced by DCA and CA in myotubes.

When these results are contextualised under pathological conditions, such as cholestasis-induced sarcopenia, it is plausible that mitochondrial dysfunction could be a mechanism involved in muscle alterations, which we observed in a murine model of cholestasis-induced sarcopenia [29, 53]. The present study also showed decreased OCR and OXPHOS complexes I, II and III in mice with cholestasis-induced sarcopenia. These findings are directly correlated with muscle strength but inversely correlated with BA levels, which supports our previous findings that BA-induced sarcopenic-like phenotype [4], suggesting that BA could be a soluble signal that negatively communicates the liver and muscle during cholestasis, thus generating muscle atrophy, weakness and energetic imbalance leading to sarcopenia. Nonetheless, it is essential to note that the effects found in this study were limited to fast-twitch muscles, which raises the question of what might occur in slow-twitch muscles. Based on the literature, a differential effect could be expected because slow fibers have high resistance to mitochondrial alterations. For example, they have a much more resistant mtROS scavenger system, which slows the progression of mitochondrial alterations [54].

In our study, a relevant finding was that DCA and CA treatments induced an increased LC3II/LC3I ratio but a decreased autophagic flux, indicating that DCA and CA caused lysosomal-dependent degradation that was lower than the basal condition. This result was reinforced by the accumulation of mitophagosome-like structures induced by the DCA and CA treatments. In the context of our study, these results could suggest that under cholestatic conditions with high BA levels, there are fewer mitochondria that present malfunction and could follow the mitophagy pathway because they are arrested in the mitophagosome formation. At the mechanistic level, the antecedents explain our results of decreased autophagic flux. Thus, BA can alter autophagic degradation by impairing autophagosomal–lysosomal fusion, which is likely due to the reduction in adaptor proteins, such as Rab7, targeting it to autophagosomes [55]. In addition, the lower lysosomal-dependent degradation of autophagosomes aligned with the unchanged mitophagy observed under the DCA and CA treatments.

Interestingly, oxidative stress is linked to alterations in mitochondrial biogenesis, mitophagy, fission and fusion [47]. Thus, the contribution of mtROS to oxidative stress in skeletal muscle under exposure to BA could explain the decreased biogenesis and alterations in autophagic flux found in the present study. These results suggest the accumulation of dysfunctional mitochondria with reduced ATP generation, supported by the reduction in the ATP-linked OCR and respiratory complexes I and II, which could be a central factor in the loss of strength and changes in fiber types observed in BA-induced sarcopenia or cholestatic liver disease [4, 29, 53].

## Conclusion

**Fig. 9:**
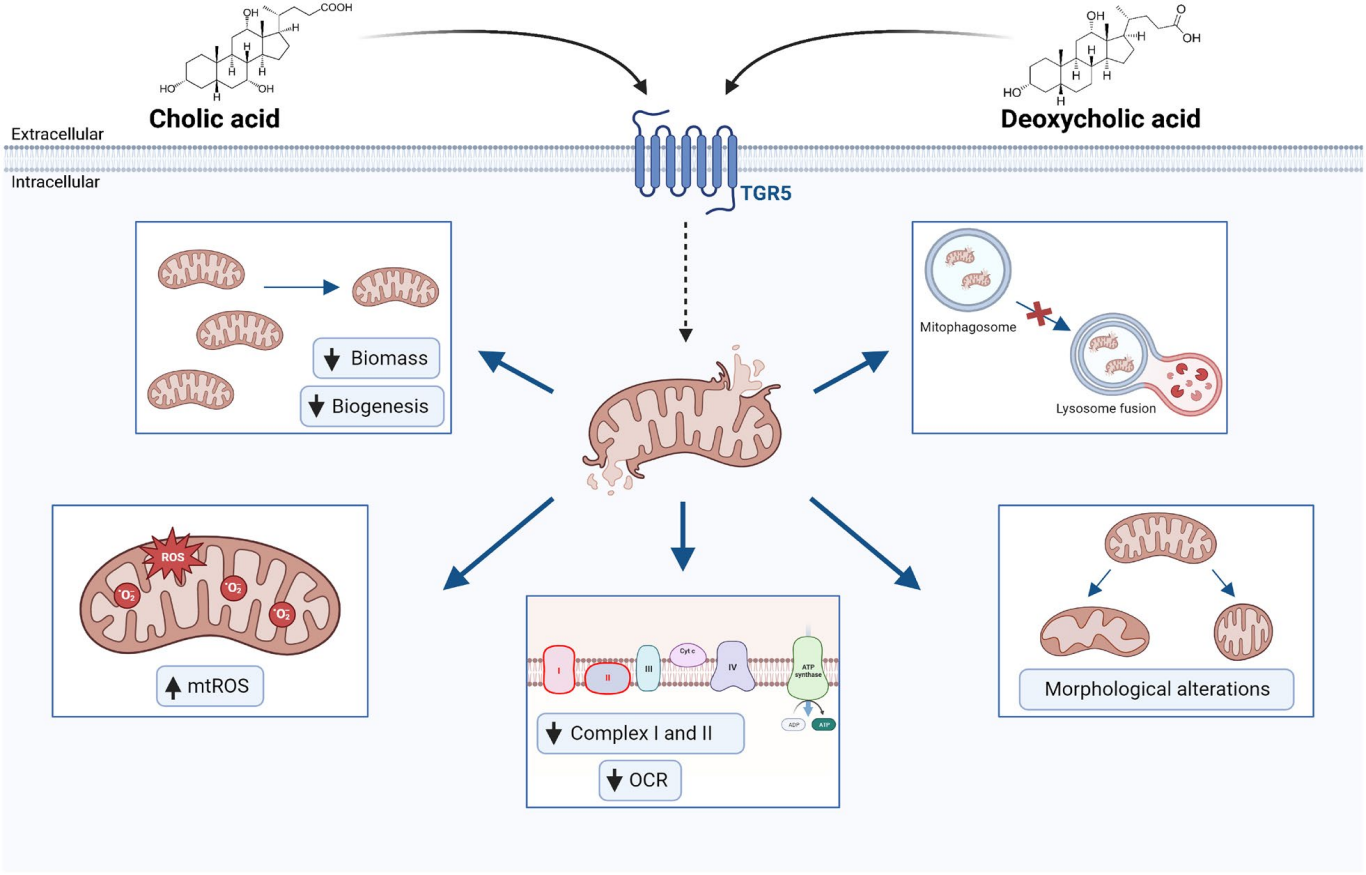
Cholic and deoxycholic acids induced mitochondrial dysfunction. Bile acids (BA) can interact with skeletal muscle cells, presumably through the TGR5 receptor, altering mitochondrial function. We demonstrated that BA diminish the mitochondrial mass in a mechanism associated with mitochondrial biogenesis decreasing. Also, we observed alterations in autophagic flux that trigger in accumulation of mitophagosome-like structures. Besides, we show that BA induce mitochondrial structural alterations, such as increased circularity and decreased cristae, that could cause impairment in mitochondrial function. These changes can be associated with less ORC and correlated with complex I and II diminutions. Finally, we demonstrated that mitochondrial oxidative stress generation in skeletal muscle cells is an important mechanism related to mitochondrial dysfunction

This study is the first to show that CA and DCA induced mitochondrial dysfunction in skeletal muscle cells, characterised by decreased mitochondrial biomass, biogenesis, bioenergetics activity and cristae number, as well as increased circularity and mtROS levels. Interestingly, we also observed the detention of autophagic flux and the accumulation of mitophagosome-like structures. Some mitochondrial alterations were also maintained in a mouse model of cholestasis-induced sarcopenia characterised by increased levels of BA, such as DCA and CA (Fig. 9).

## Methods

### Cell cultures

As previously described, the skeletal muscle cell line C_2_C_12_ (American Type Culture Collection, ATCC, Manassas, VA, USA) was grown and differentiated until day 5 to obtain myotubes [56, 57]. The myotubes were incubated with deoxycholic acid (DCA) and cholic acid (CA) (Sigma-Aldrich, St. Louis, MO, USA) at 120 and 500 μM, respectively, for the times indicated in each figure. DCA and CA are the most critical BA present in the cholestatic conditions of chronic liver diseases. The concentrations used in this study are according to circulating levels observed in this condition and were probed to maintain the cell viability in our previous report [4, 58].

### Animals

16 weeks old C57BL/6 male mice were randomized and separated to perform three independent experiments. Mice were fed with a standard diet or supplemented with 0.1% 5-diethoxycarbonyl-1,4-dihydrocollidine (DDC) (Sigma-Aldrich, St. Louis, MO, USA) for 6 weeks [29, 37]. The TA muscles were dissected and stored at – 80 ℃. Each experimental group contained 5–7 mice. Animal handling and treatments followed international, national, and institutional suggestions explicit in animal care and use guidelines. The procedures with animals had the Animal Ethics Committee’s approval at the Universidad Andrés Bello (approval number 004/2020).

### Grip strength test

The grip strength test was done according to the previously published protocol to assess the maximum force on the hindlimb [29, 37]. Briefly, 15 repetitions of force were performed on the hind legs, and the three best values were recorded. The values were normalized by the animal weight.

### Plasma bile acid levels

Blood obtained from the mouse’s tail was centrifuged at 1500 × g for 10 min at 4 ℃. The plasma was used to determine total bile acid levels using a colorimetric assay (Randox Laboratories Ltd., Kearneysville, WV, USA) [37]. The samples were measured at 405 nm after 60 and 120 s (S1 and S2, respectively). The difference between both values was calculated (ΔS), and the plasma bile acid concentration was calculated by the following formula:

$$\left( {{\raise0.7ex\hbox{${\Delta S sample}$} \!\mathord{\left/ {\vphantom {{\Delta S sample} {\Delta S calibrator}}}\right.\kern-0pt} \!\lower0.7ex\hbox{${\Delta S calibrator}$}}} \right) \cdot \left[ {calibrator} \right] = \left[ {sample} \right] \mu mol/L$$

### Muscle fiber isolation

Myofibers were isolated from EDL muscles of C57BL/6 and treated with a standard and DDC-supplemented diet for 6 weeks. EDL muscle was dissected and digested in an F12 medium (Life Technologies, Inc, Carlsbad, CA, USA) using 750 U/ml collagenase type I (Worthington Biochemical Corporation, Lakewood, NJ, USA) at 37 °C for 1 h. Then, myofibers were dissociated by flushing the muscle with F12‐15% horse serum. After, myofibers were extracted individually using a dissecting microscope and fire‐polished pipettes. Approximately 20–50 myofibers were transferred into 24‐well plates covered with Matrigel Matrix GFR (Corning, Glendale, AZ, USA) (1:1 dilutes in F12) and incubated overnight in incubator conditions (37 °C and 5% CO2) [4].

### Western blot analysis

For the protein extracts, myotubes or TA muscles were homogenized in a radioimmunoprecipitation assay (RIPA) buffer with 1 mM of a cocktail of protease inhibitors (Sigma-Aldrich, St. Louis, MO, USA) and 1 mM of phenylmethylsulfonyl fluoride (Sigma-Aldrich, St. Louis, MO, USA). Proteins were subjected to SDS-PAGE, transferred onto polyvinylidene difluoride membranes (Thermo Fisher Scientific, Waltham, MA, USA), and probed with anti-OXPHOS (1:1000; Abcam, Cambridge, MA, USA), anti-PGC-1α (1:1000, Cell Signaling, Danvers, MA, USA), anti-PINK-1 (1:1000, Cell Signaling, Danvers, MA, USA), anti-LC3B (1:1000, Cell Signaling, Danvers, MA, USA), anti-TOM20 (1:1000, Cell Signaling, Danvers, MA, USA), anti-GAPDH (1:2000; Santa Cruz, Dallas, TX, USA) and anti-β-actin (1:1000; Abcam, Cambridge, MA, USA) antibodies. All immunoreactions were visualized by enhanced chemiluminescence (Thermo Scientific, Waltham, MA, USA). Images were acquired using the Fotodyne FOTO/Analyst Luminary Workstation Systems (Fisher Scientific, St. Waltham, MA, USA). Densitometry analysis was determined by scanning immunoreactive bands. Intensity values were obtained for further normalization against the control group using ImageJ software (National Institutes of Health [NIH], Bethesda, MD, USA).

### Autophagy assay

The protein levels of LC3I and LC3II were detected by Western blot. The LC3II/LC3I ratio was analyzed as a parameter of autophagy. Autophagic flux was determined by analysis of the difference between LC3II protein levels in cells incubated in the presence and absence of 50 μM of chloroquine (CQ) (Sigma-Aldrich, St Louis, MO, USA), as described previously [59].

### Immunofluorescence microscopy

The levels of mitochondrial protein TOM20 were analyzed by indirect immunofluorescence. Briefly, cells were grown and differentiated on glass coverslips and then fixed in 4% paraformaldehyde, permeabilized with 0.05% Triton X-100, and incubated for 1 h with 1:100 rabbit anti-TOM20 (Cell Signaling, Danvers, MA, USA) in a buffer containing 50 mM Tris–HCl, pH 7.7; 0.1 M NaCl; and 2% bovine serum albumin. After antibody removal and several washes with the mentioned buffer, bound antibodies were detected by incubating the cells for 30 min with 1:100 AlexaFluor 488 anti-rabbit as the secondary antibody (Thermofisher Scientific, Waltham, MA USA). Images were captured using a confocal microscope TCS SP8 (Leica, Wetzlar, Germany) and analyzed using ImageJ software (NIH, Bethesda, MD, USA). Three independent experiments were quantified with four fields of view (areas) analyzed in 25–30 myotubes per condition per each separate experiment.

### Transmission electron microscopy

Myotubes were treated with DCA and CA. After 72 h, cells were fixed in a glutaraldehyde 4% solution. Three washes with sodium cacodylate buffer 0.1 M were made before 2 h of osmium tetroxide 2% staining. Also, a second staining was done for 2 h with uranyl acetate and posterior washes with 1% cacodylate buffer. Dehydration with acetone gradients was made, and myotubes were embedded in Epon [60, 61]. 80-nm sections were mounted on electron microscopy grids for examination using a transmission electron microscope (Philips, Tecnai 12 at 80 kV). For each independent experiment was analyzed the cell mitochondria of at least three fields in four myotubes and were counting 6–8 mitochondria per field. The mitochondrial density was calculated by the mitochondrial percentage of the total area, and the mitochondrial size was determined in nm^2^. The width/lengthy ratio was calculated to obtain the mitochondrial circularity. The mitochondrial cristae number was normalized by the mitochondrial size. Finally, the number of mitophagosome-like structures was counted manually. An expert made all quantifications in a blind fashion at 20,000 × magnification.

### Mitochondrial morphology

To evaluate mitochondrial morphology, a Mitotracker Red CMXROS probe (Thermofisher Scientific, Waltham, MA, USA) was used. C_2_C_12_ myotubes treated with DCA and CA for 72 h were washed with Hank’s balanced salt solution (HBSS) and incubated with a Mitotracker Red CMXROS Probe (20 nM) in HBSS for 30 min at 37 °C, 5% CO2. Then, the myotubes were washed with HBSS and fixed with 4% paraformaldehyde. Images were captured using a confocal microscope TCS SP8 (Leica, Wetzlar, Germany) and analyzed using the software ImageJ (NIH, Bethesda, MD, USA) with the tool Mitochondrial Network Analysis (MiNA). Three independent experiments were quantified with four fields of view (areas) analyzed in 25–30 myotubes per condition per each separate experiment.

### Mitochondrial potential

The mitochondrial potential was evaluated using a Tetramethylrhodamine, ethyl ester (TMRE) probe (Thermofisher Scientific, Waltham, MA, USA). C_2_C_12_ myotubes were treated with DCA, CA, or vehicle for 72 h. At the end of the experiments, they were incubated with 10 nM of TMRE (no-quenching mode) at 37 °C in a 5% CO2 incubator for 30 min. Furthermore, images were captured in live myotubes using a confocal microscope TCS SP8 (Leica, Wetzlar, Germany) and analyzed using ImageJ software (NIH, Bethesda, MD, USA) with the MiNA tool to calculate elongated and round mitochondria.

Three independent experiments were quantified with four fields of view (areas) analyzed in 25–30 myotubes per condition per each separate experiment.

### Measurement of mitochondrial ROS levels

C_2_C_12_ myotubes were treated with DCA, CA, or vehicle for 72 h. At the end of the experiments, they were incubated with 5 μM Mitosox Red (Thermofisher Scientific, Waltham, MA, USA) at 37 °C in a 5% CO2 incubator for 30 min. Mitosox is a fluorogenic dye targeted explicitly to mitochondria in live cells. Its oxidation by superoxide produces red fluorescence. Images were captured using a confocal microscope TCS SP8 (Leica, Wetzlar, Germany) and analyzed using ImageJ software with the MiNA tool. Mitosox Red is a probe whose entrance to the mitochondria depends on the potential. For this reason, the fluorescence intensity of Mitosox was normalized with the TMRE signal. Three independent experiments were quantified with four fields of view (areas) analyzed in 25–30 myotubes per condition per each separate experiment.

Complementary mtROS levels were also measured by flow cytometry. After 72 h treatment with DCA or CA, a wash with DMEM was performed, and the myotubes were incubated with 10 µM of MitoSOX Red probe in DMEM 4% SC for 30 min. Then, two washes were performed, with DMEM and PBS, and the cell was incubated with trypsin–EDTA (Thermofisher Scientific, Waltham, MA, USA) for 7 min. 500 µL of DMEM was aggregated, and with the help of a scrapper, the cells were collected in a 1.5 mL Eppendorf tube and centrifuged at 2000 × g for 10 min. The supernatant was discarded, and the pellet was resuspended in 300 µL of PEB buffer (PBS-EDTA 2 mM-BSA 0.5%). Finally, the cells were analyzed by flow cytometry using an LSRFortessa X20 equipment (BD Biosciences, San Jose, CA, USA). The mean fluorescence intensity (MFI) was determined in the samples after acquiring 100,000 total events. The MFI was normalized by cell number, quantified for each condition, and compared to the control condition.

### Analysis of cellular respiration and mitochondrial energetics

C_2_C_12_ myotubes or isolated EDL muscle fibers were seeded in XF24-3-well microplates (Seahorse Bioscience, Billerica, MA, USA) covered with Matrigel Matrix GFR (Corning, Glendale, AZ, USA). Only the myotubes were incubated with DCA and CA for 72 h. Then, both samples were maintained without CO2 for 1 h. The XF assay medium was low-buffered bicarbonate-free Dulbecco's Modified Eagle Medium (DMEM) (pH 7.4) and replicated the glucose and pyruvate/glutamax composition of the respective experimental conditions. Analysis of oxygen consumption rate (OCR) was evaluated at different stages: basal respiration, ATP-linked respiration, H^+^-leak, maximal respiratory capacity, spare respiratory capacity, and non-mitochondrial respiration using modulators of cellular respiration: 1 μM oligomycin, 1 μM carbonyl cyanide p-trifluoromethoxyphenylhydrazone (FCCP), rotenone and antimycin A, as previously described [62, 63]. Besides, Seahorse Bioscience XF24-3 Extracellular Flux Analyzer (Agilent, Santa Clara, CA, USA) was used to evaluate the mitochondrial parameters normalized to protein content/well within the Seahorse plate. For Seahorse XF analyzer studies, data points per experimental condition were collected from a minimum of three replicates, with each experiment being conducted at least three times. After detection, the cellular protein content was quantitated with a MicroBCA kit (Thermofisher Scientific, Waltham, MA USA), and OCR was normalized accordingly.

### Fluorescence imaging-based analysis of mitophagy

Myoblasts were seeded in DMEM 10% SFB medium on a glass-cell culture plate (SPL cell culture slide 2W) coated with Matrigel Matrix GFR (Corning, Glendale, AZ, USA) until differentiation to myotubes. Then, myotubes were treated with 120 µM DCA or 500 µM CA for 48 h. After the treatment, myotubes were washed with DMEM, 500 nM Mitotracker Green FM (Thermofisher Scientific, Waltham, MA USA), and 25 nM LysoTracker Red DND-99 (Thermofisher Scientific, Waltham, MA USA) mixture probes was added, incubating them for 30 min. At the end of incubation, two washes with DMEM were carried out, and images were acquired using a TCS SP8 confocal fluorescence microscope (Leica, Wetzlar, Germany). The following two laser wavelengths were used FITC (Ex 488 nm and Em 560–727 nm), and Alexa Fluor 647 (Ex 638 nm and Em 794–799 nm), and signals were detected with an ultrahigh dynamic photomultiplier (PMT) spectral detector. The co-localization of labeled mitochondria and lysosomes was used as a mitophagy process marker. For this, the JACoP plugins of the ImageJ (NIH, Bethesda, MD, USA) software were used to determine the Manders’ coefficient [64], which indicates overlapping of the red signal (Lysotracker) on the green signal (Mitotracker), where 0 corresponds to signals that do not overlap, and 1 represents 100% overlap between both signals. Three independent experiments were quantified with four fields of view (areas) analyzed in 25–30 myotubes per condition per each separate experiment, taking an intermediate z-stack for each field.

### PGC-1α plasmid reporter activity

1 µg of PGC-1α promoter 2 Kb luciferase (gift from Bruce Spiegelman. Addgene plasmid # 8887), 0.02 μg of pRL-SV40 (gift from Ron Prywes. Addgene plasmid # 27163) were used to co-transfect cells using 1 μl of LipofectAMINE 3000 (Thermofisher Scientific, Waltham, MA USA) in Opti-MEM I (Thermofisher Scientific, Waltham, MA USA). After 6 h, FBS was added to the medium, and the cells were cultured for 12 h. Further, the cells were differentiated for 4 days, and myotubes were incubated with DCA (120 μM) and CA (500 μM) for 24 h. Dual luciferase activity assays (Promega, Madison, WI, USA) were performed in a GloMax 20/20 luminometer (Promega, Madison, WI, USA). The Renilla luciferase activity value normalized each luciferase activity value.

### Mitochondrial DNA quantification

C_2_C_12_ myotubes were incubated with DCA, CA, or vehicle for 72 h. After each treatment, total DNA extraction was performed. The DNA amount was quantified by spectrophotometry. The mitochondrial DNA (mtDNA) was detected and quantified by qPCR using primers to recognize the mitochondrial *Cytb* gene (*mt-Cytb*, forward: 5′-CCATTCTACGCTCAATCCCCAATA-3′; and reverse: 5′-CTACTGGTTGGCCCCCAATTC-3′). The mtDNA levels were normalized by the nuclear gene *B2m* (forward: 5′-GGGTCATGGTCTGTGAAGCA-3′; and reverse: 5′-CAGAGGCTCTATCGCGGAAA-3′), which were detected by qPCR using the SyBR Green method. PCR was performed in triplicate using an Eco Real-Time PCR System (Illumina, San Diego, CA, USA).

### Statistics

The statistical significance of differences between the means of the experimental groups was evaluated using two-tailed T-tests (Prism 9.0, GraphPad, San Diego, CA, USA). A difference was considered statistically significant at a p-value < 0.05.

## Supplementary Information

**Additional file 1: **The increased mitochondrial punctate pattern is induced by DCA and CA in C2C12 myotubes. C2C12 myoblasts were differentiated for 5 days until forming myotubes. Then, cells were incubated with 120 μM of DCA or 500 μM of CA for 72 h. Further, myotubes were incubated with a Mitotracker red probe for 30 min. (a) Representative images for the Mitotracker red signal (standard size: upper panel; magnification zoom: lower panel). Scale bar: 10 μm. (b-c) The Mitotracker red-based punctate pattern was analyzed in myotubes incubated with DCA (b) or CA (c). Values are expressed as a fold of induction and correspond to the mean ± SEM (n = 3 independent experiments, *p < 0.05. t-test).

**Additional file 2: **DCA and CA decrease PINK-1 levels in C2C12 myotubes. C2C12 myoblasts were differentiated for 5 days until forming myotubes. Then, cells were incubated with 120 μM of DCA or 500 μM of CA for 72 h. (a) Protein levels of PINK-1 were detected by Western blot analysis using β-actin levels as a loading control. Molecular weight markers are depicted in kDa. The quantitative analysis of value is expressed as a fold of change for DCA (b) and CA (c). Values correspond to the mean ± SEM (n = 3 independent experiments, *p < 0.05. t-test).

**Additional file 3: **Decreased muscle strength correlates with bile acids in a mouse model of cholestasis-induced sarcopenia. C57BL/6 mice were fed with a chow or a DDC-supplemented diet for 6 weeks. (a) The hindlimb grip test was performed at the end of the treatments to measure the strength and normalize by body weight. (b) Plasma bile acid levels were determined at the end of the diet and expressed as fold of change concerning the chow diet. The values correspond to the mean ± SEM (n = 5-7 animals per condition, *p < 0.05 vs chow diet. t-test). (c) Heatmap of Pearson correlation for bile acids levels and strength represents the relation between both variables.

## Data Availability

The datasets used and/or analyzed during the current study are available from the corresponding author upon reasonable request.

## Abbreviations

BA: Bile acids
CA: Cholic
Chow: Standard diet
DCA: Deoxycholic
DDC: Hepatotoxin
EDL: Extensor digitorum longus
FCCP: Carbonyl cyanide p-trifluoromethoxyphenylhydrazone
LC3B: Microtubule-associated proteins 1A/1B light chain 3B
mtROS: Mitochondrial reactive oxygen species
OCR: Oxygen consumption rate
OXPHOS: Oxidative phosphorylation
PGC-1α: Peroxisome proliferator-activated receptor gamma coactivator 1-alpha
TA: Tibialis anterior
TLCA: Taurolithocholic acid
UDCA: Ursodeoxycholic acid
TGR5: Takeda-G-protein-receptor-5
TMRE: Tetramethylrhodamine ethyl ester
TOM20: Translocase of outer mitochondrial membrane 20
TFAM: Mitochondrial transcription factor A
PINK-1: PTEN Induced Kinase 1
mTOR: mammalian target of rapamycin
AMPK: AMP-activated protein kinase
ETC: Electron transport chain
ROS: Reactive oxygen species
