# Supplementary figures and images for: Cholic and deoxycholic acids induce mitochondrial dysfunction, impaired biogenesis and autophagic flux in skeletal muscle cells
Source: Biol Res. 2023 Jun 8;56:30. doi: 10.1186/s40659-023-00436-3 (PMC10249330; doi:10.1186/s40659-023-00436-3)

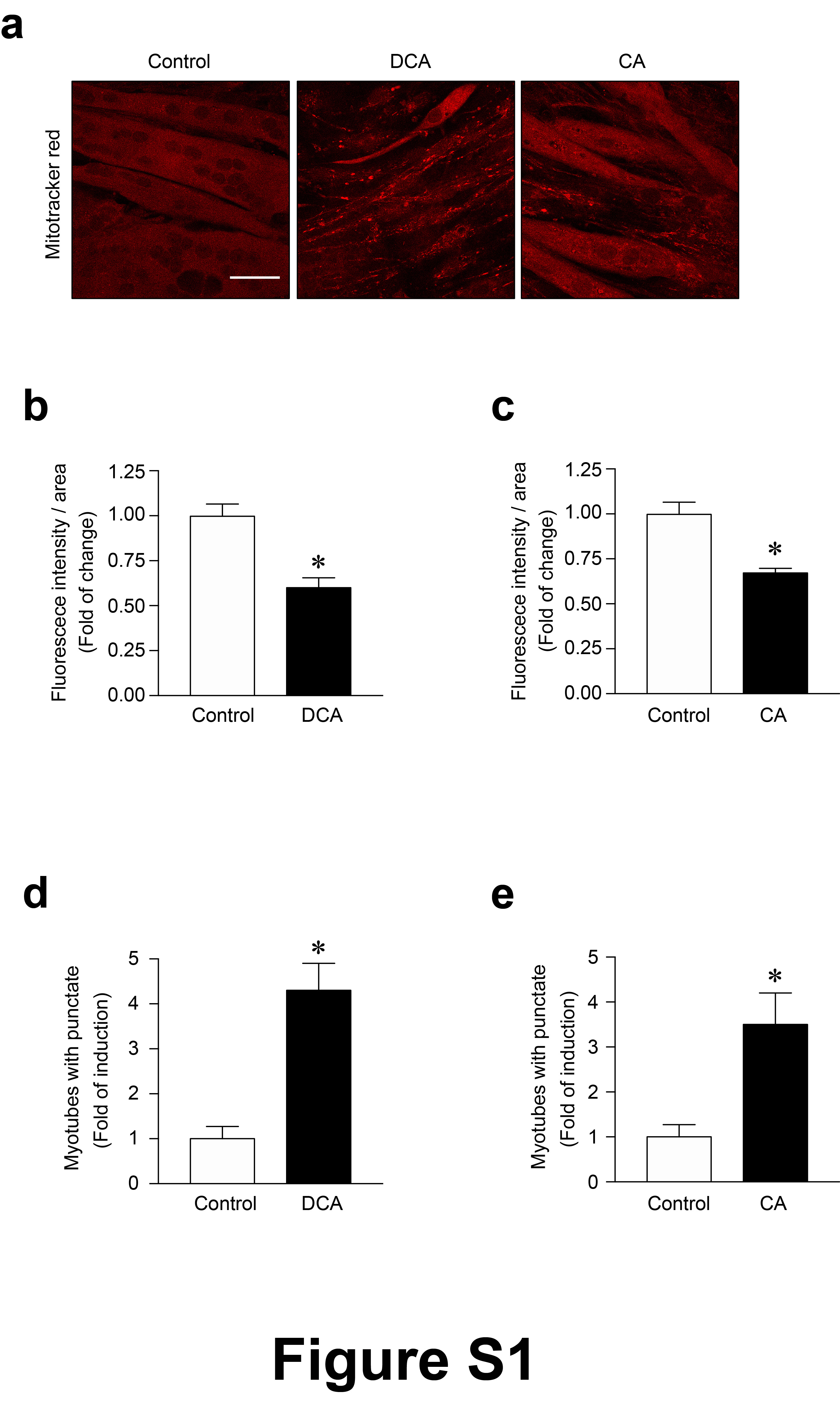

Supplement: Supplementary file 1 — Additional file 1: The increased mitochondrial punctate pattern is induced by DCA and CA in C2C12 myotubes. C2C12 myoblasts were differentiated for 5 days until forming myotubes. Then, cells were incubated with 120 μM of DCA or 500 μM of CA for 72 h. Further, myotubes were incubated with a Mitotracker red probe for 30 min. (a) Representative images for the Mitotracker red signal (standard size: upper panel; magnification zoom: lower panel). Scale bar: 10 μm. (b-c) The Mitotracker red-based punctate pattern was analyzed in myotubes incubated with DCA (b) or CA (c). Values are expressed as a fold of induction and correspond to the mean ± SEM (n = 3 independent experiments, *p < 0.05. t-test). [file 40659_2023_436_MOESM1_ESM.jpg]

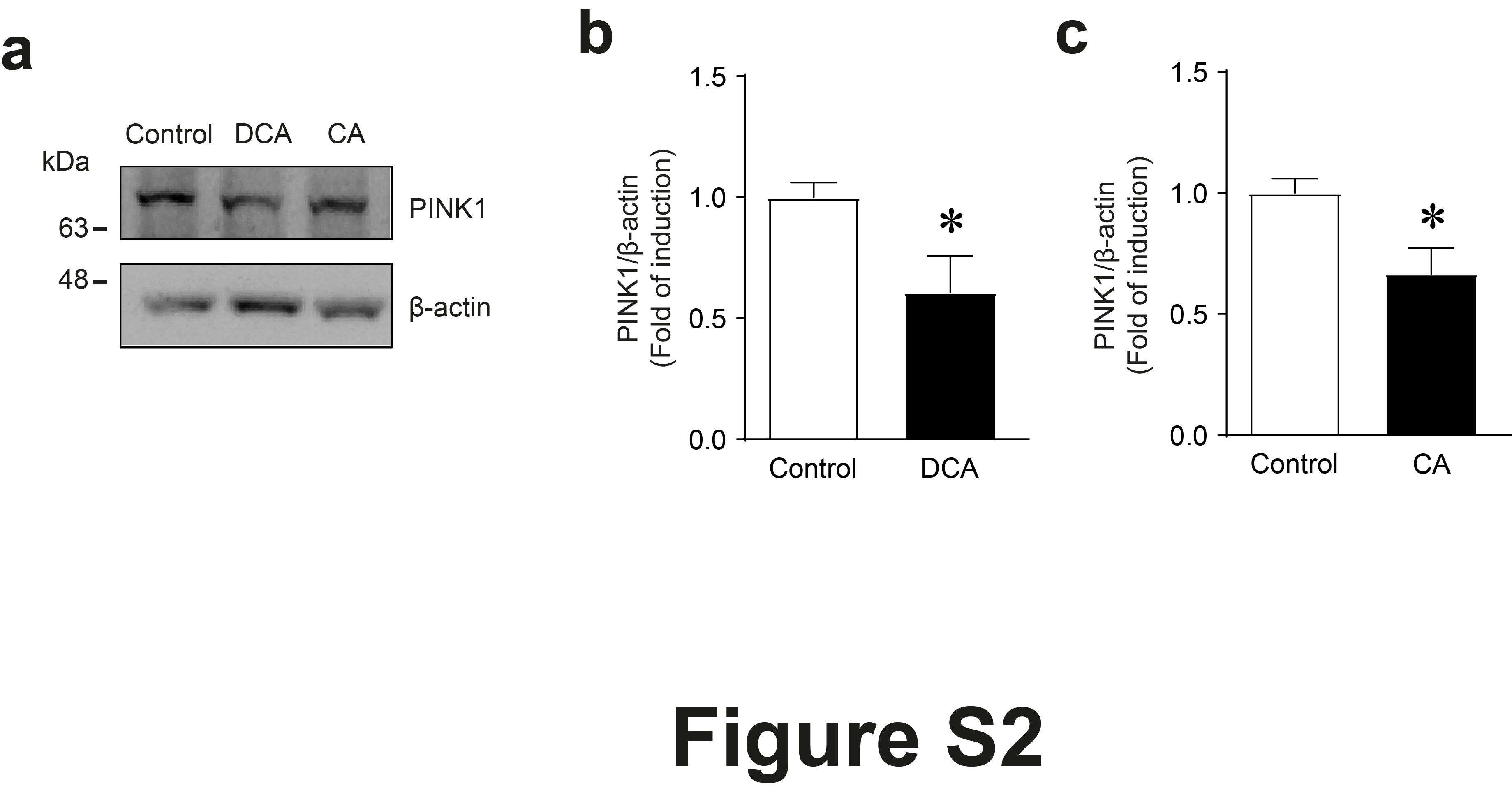

Supplement: Supplementary file 2 — Additional file 2: DCA and CA decrease PINK-1 levels in C2C12 myotubes. C2C12 myoblasts were differentiated for 5 days until forming myotubes. Then, cells were incubated with 120 μM of DCA or 500 μM of CA for 72 h. (a) Protein levels of PINK-1 were detected by Western blot analysis using β-actin levels as a loading control. Molecular weight markers are depicted in kDa. The quantitative analysis of value is expressed as a fold of change for DCA (b) and CA (c). Values correspond to the mean ± SEM (n = 3 independent experiments, *p < 0.05. t-test). [file 40659_2023_436_MOESM2_ESM.jpg]

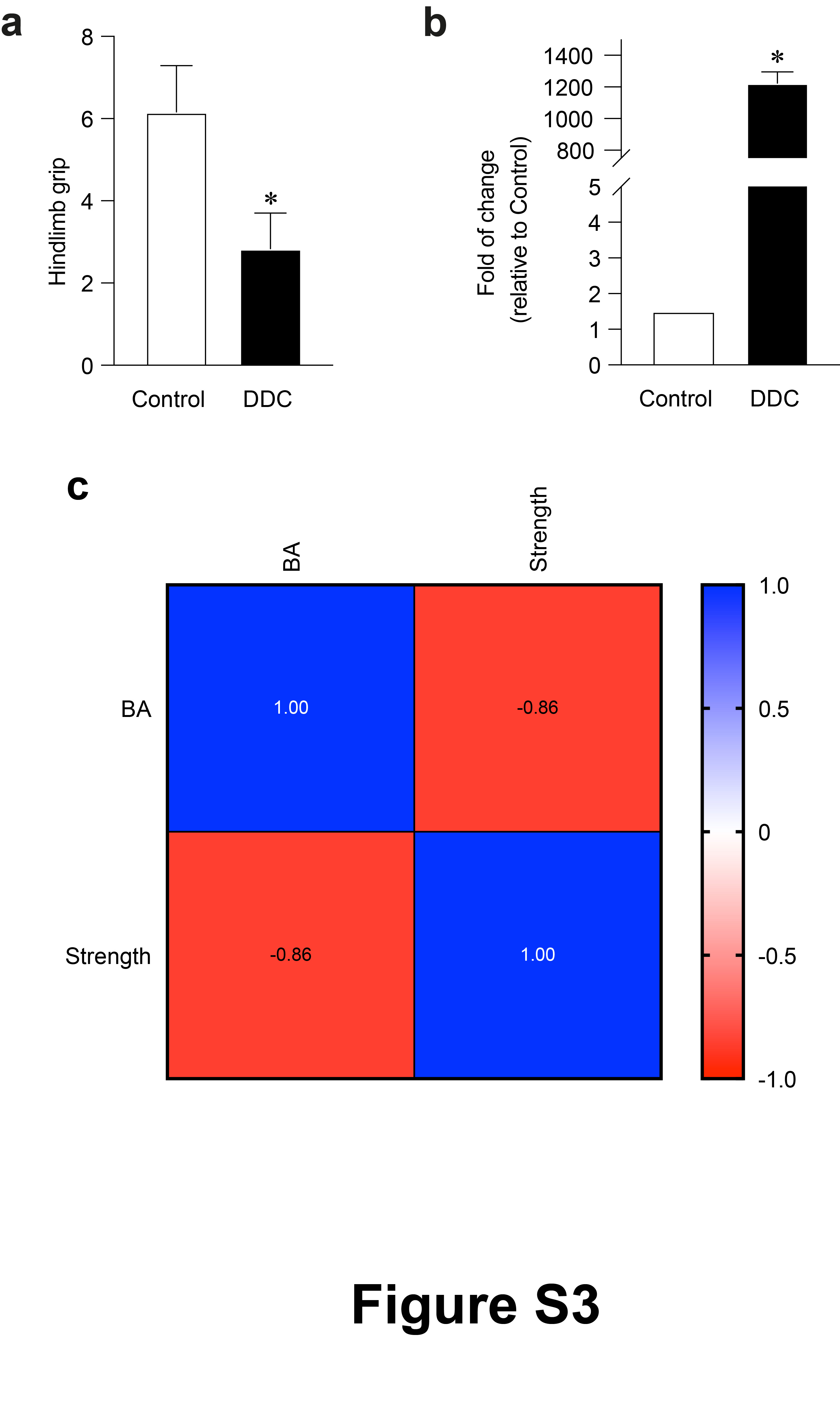

Supplement: Supplementary file 3 — Additional file 3: Decreased muscle strength correlates with bile acids in a mouse model of cholestasis-induced sarcopenia. C57BL/6 mice were fed with a chow or a DDC-supplemented diet for 6 weeks. (a) The hindlimb grip test was performed at the end of the treatments to measure the strength and normalize by body weight. (b) Plasma bile acid levels were determined at the end of the diet and expressed as fold of change concerning the chow diet. The values correspond to the mean ± SEM (n = 5-7 animals per condition, *p < 0.05 vs chow diet. t-test). (c) Heatmap of Pearson correlation for bile acids levels and strength represents the relation between both variables. [file 40659_2023_436_MOESM3_ESM.jpg]
